# Beyond the exome: utility of long-read whole genome sequencing in exome-negative autosomal recessive diseases

**DOI:** 10.1186/s13073-023-01270-8

**Published:** 2023-12-14

**Authors:** Lama AlAbdi, Hanan E. Shamseldin, Ebtissal Khouj, Rana Helaby, Bayan Aljamal, Mashael Alqahtani, Aisha Almulhim, Halima Hamid, Mais O. Hashem, Firdous Abdulwahab, Omar Abouyousef, Amal Jaafar, Tarfa Alshidi, Mohammed Al-Owain, Amal Alhashem, Saeed Al Tala, Arif O. Khan, Elham Mardawi, Hisham Alkuraya, Eissa Faqeih, Manal Afqi, Salwa Alkhalifi, Zuhair Rahbeeni, Samya T. Hagos, Wijdan Al-Ahmadi, Seba Nadeef, Sateesh Maddirevula, Khalid S. A. Khabar, Alexander Putra, Angel Angelov, Changsook Park, Ana M. Reyes-Ramos, Husen Umer, Ikram Ullah, Patrick Driguez, Yoshinori Fukasawa, Ming Sin Cheung, Imed Eddine Gallouzi, Fowzan S. Alkuraya

**Affiliations:** 1 Department of Zoology, Collage of Science, King Saud University, Riyadh, Saudi Arabia; 2 Department of Translational Genomics, Center for Genomic Medicine, King Faisal Specialist Hospital and Research Center, Riyadh, Saudi Arabia; 3 Department of Medical Genomics, Center for Genomic Medicine, King Faisal Specialist Hospital and Research Center, Riyadh, Saudi Arabia; 4 Collage of Medicine, Alfaisal University, Riyadh, Saudi Arabia; 5 Pediatric Department, Division of Genetic and Metabolic Medicine, Prince Sultan Medical Military City, Riyadh, Saudi Arabia; 6 Pediatric Department, Neonatal Unit, Armed Forces Hospital, Khamis Mushayt, Saudi Arabia; 7 Eye Institute, Cleveland Clinic Abu Dhabi, Abu Dhabi, United Arab Emirates; 8 Department of Ophthalmology, Cleveland Clinic Lerner College of Medicine of Case Western Reserve University, Cleveland, OH USA; 9 Maternal Fetal Medicine, Security Forces Hospital Program, Riyadh, Saudi Arabia; 10 Vitreoretinal Surgery and Ocular Genetics, Global Eye Care/Specialized Medical Center Hospital, Riyadh, Saudi Arabia; 11 Section of Medical Genetics, King Fahad Medical City, Children’s Specialist Hospital, Riyadh, Saudi Arabia; 12 Metabolic and Genetic Center, King Salman Bin Abdulaziz Medical City, Almadinah Almunwarah, Saudi Arabia; 13 Newborn Screening, Ministry of Health, Eastern Province, Saudi Arabia; 14 Department of Clinical Genomics, Center for Genomic Medicine, King Faisal Specialist Hospital and Research Center, Riyadh, Saudi Arabia; 15 Department of Molecular Biomedicine, King Faisal Specialist Hospital and Research Centre, Riyadh, Saudi Arabia; 16 King Abdullah University of Science and Technology (KAUST), Core Labs, Thuwal, Saudi Arabia; 17 KAUST Smart-Health Initiative King Abdullah University of Science and Technology (KAUST), Thuwal, Saudi Arabia; 18 Engineering (BESE) Division, King Abdullah University of Science and Technology (KAUST), Thuwal, Saudi Arabia

**Keywords:** Long-read sequencing, Autozygome, *STX3*, *ABHD12*, *C1orf109*, *FLVCR1*, *NID1*, *PKHD1*, SHFM

## Abstract

**Background:**

Long-read whole genome sequencing (lrWGS) has the potential to address the technical limitations of exome sequencing in ways not possible by short-read WGS. However, its utility in autosomal recessive Mendelian diseases is largely unknown.

**Methods:**

In a cohort of 34 families in which the suspected autosomal recessive diseases remained undiagnosed by exome sequencing, lrWGS was performed on the Pacific Bioscience Sequel IIe platform.

**Results:**

Likely causal variants were identified in 13 (38%) of the cohort. These include (1) a homozygous splicing SV in *TYMS* as a novel candidate gene for lethal neonatal lactic acidosis, (2) a homozygous non-coding SV that we propose impacts *STK25* expression and causes a novel neurodevelopmental disorder, (3) a compound heterozygous SV in *RP1L1* with complex inheritance pattern in a family with inherited retinal disease, (4) homozygous deep intronic variants in *LEMD2* and *SNAP91* as novel candidate genes for neurodevelopmental disorders in two families, and (5) a promoter SNV in *SLC4A4* causing non-syndromic band keratopathy. Surprisingly, we also encountered causal variants that could have been identified by short-read exome sequencing in 7 families. The latter highlight scenarios that are especially challenging at the interpretation level.

**Conclusions:**

Our data highlight the continued need to address the interpretation challenges in parallel with efforts to improve the sequencing technology itself. We propose a path forward for the implementation of lrWGS sequencing in the setting of autosomal recessive diseases in a way that maximizes its utility.

**Supplementary Information:**

The online version contains supplementary material available at 10.1186/s13073-023-01270-8.

## Background

Mendelian diseases are defined by high-impact variants in single genes that are typically sufficient to cause the phenotype. The identification of these variants, therefore, represents an important diagnostic step that enables such important clinical actions as accurate counseling, reproductive planning, prognostication, and treatment. Variant identification, however, was a daunting task until the recent advent of next-generation sequencing, which allowed for exome-wide or genome-wide interrogation of candidate variants even when the correct clinical diagnosis (i.e., based on the phenotype only) is lacking [1]. The average diagnostic yield of exome and genome sequencing is typically < 50% although that varies by clinical indication [2].

The focus of exome sequencing on the coding part of the genome prompted many to investigate the diagnostic contribution of the non-coding genome using whole genome sequencing [3–5]. Surprisingly, however, the added diagnostic value of genome over exome is only modest, and even then, most of the additional molecular diagnoses were actually identifiable by exome [3, 4, 6]. This suggests that interpretation challenges remain an important factor contributing to negative exomes and that expanding the coverage using the same short-read sequencing technology is unlikely to fully capture the missing variants. Indeed, we have previously shown using positional mapping, which is impartial to variant class, that at least in autosomal recessive diseases in consanguineous families, short-read exome sequencing should in theory uncover > 90% of the underlying variants and that this hypothetical yield is not attained in practice primarily due to interpretation challenges [7, 8]. This has subsequently been borne out by detailed analysis of > 4500 molecularly characterized families in which we specifically explored different types of interpretation challenges and how the special characteristics of our highly consanguineous population can help address them [9].

There are two major long-read sequencing (lrWGS) technologies in the market: single molecule real-time (SMRT) sequencing by Pacific Biosciences (PacBio) and nanopore sequencing by Oxford Nanopore Technologies Inc. (ONT) [10, 11]. Despite their availability for more than a decade, their clinical use has been eclipsed by the very widespread use of short-read sequencing that continues to dominate the diagnostic landscape. Several attempts, however, have been made to show the added value of lrWGS over srWGS and WES in diagnostically challenging cases. For example, Borras et al. have shown in 2017 the value of targeted long-read sequencing in the challenging *PKD1* locus [12]. In 2018, Merker et al. performed the first lrWGS in a patient with a suspected Mendelian disorder and identified a SV in *PRKAR1A* as a candidate cause of their patient’s Carney complex [13]. In the same year, Sanchis-Juan et al. also deployed lrWGS in a single patient to reveal a de novo duplication-inversion-duplication overlapping *CDKL5* [14]. A larger cohort of 40 patients was reported by Miller et al.; however, they used targeted long-read sequencing rather than WGS because they only included cases with known candidate copy number variant (CNV) or gene a priori [15]. Thus, there remains an unmet need to evaluate the added value of lrWGS over exome sequencing in the diagnostic workup of patients with suspected Mendelian disorders [16].

In this study, we aim to test the hypothesis that when interpretation challenges are adequately addressed and excluded in exome sequencing, lrWGS should be used for the diagnostic workup. Specifically, such cases should be enriched for variant classes that are not readily discoverable by short-read exome sequencing such as structural variants (SV), repeats, and non-coding variants.

## Methods

### Human subjects

**Fig. 1:**
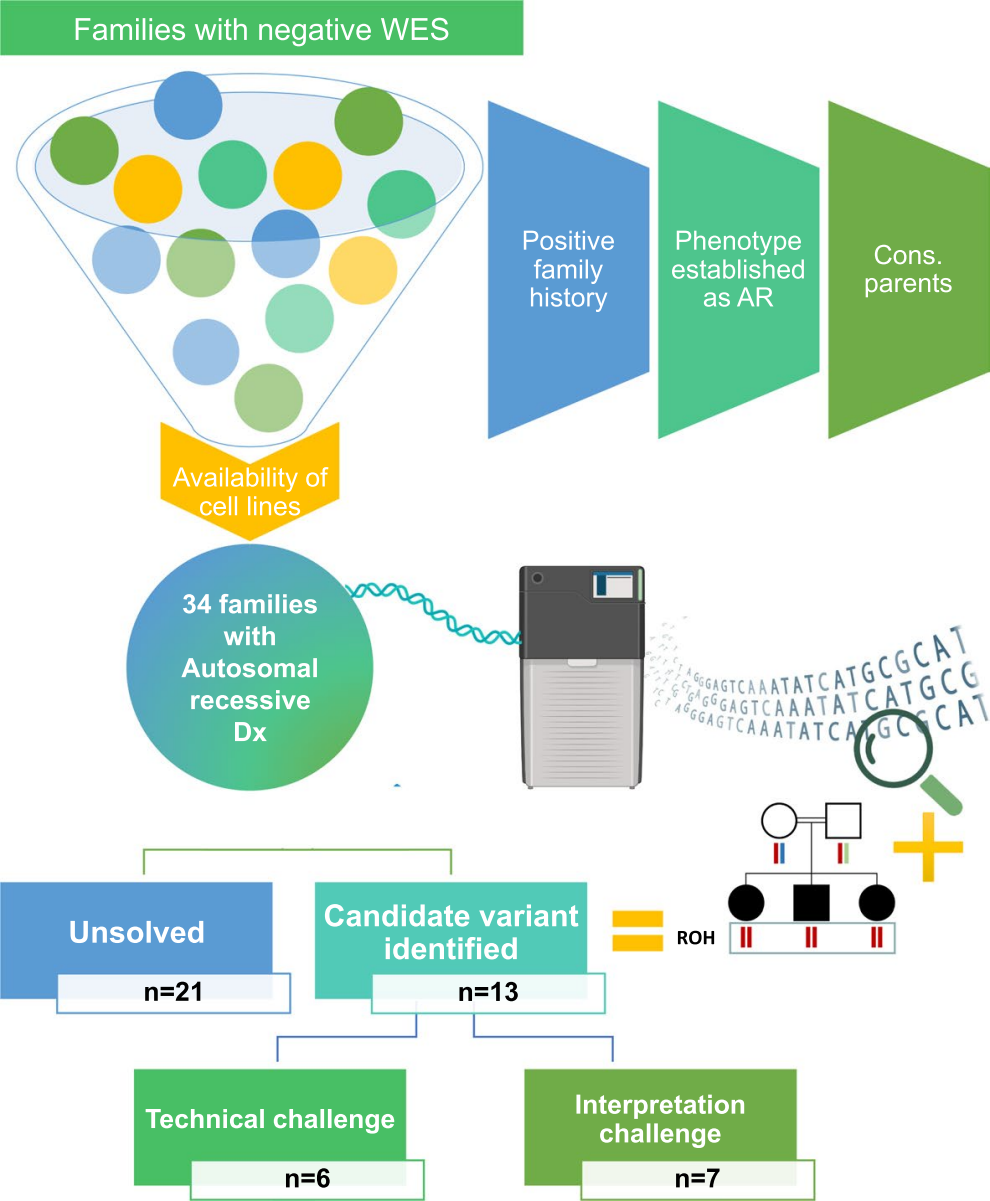
Schematic presentation of the study design. All cases with negative exome reanalysis with available cell lines were considered if they met at least two of the following criteria: the case has a positive family history for the same disease, the phenotype is established as autosomal recessive, and the parents are consanguineous. Ultramolecular weight DNA was extracted from established cell lines for each case and underwent lrWGS using PacBio technology. Autozygome-guided analysis was performed, and candidate variants were identified in 13 families. Six variants were not identified by exome or its reanalysis and presented technical challenges while the remaining seven variants presented interpretation challenges. Cons: consanguineous. Some illustrations were created using BioRender.com

A total of 34 families of Middle Eastern ancestry were recruited after obtaining informed consent under IRB-approved research protocols (RAC# 2121053, 2080006, 2070023, 2210029). Detailed phenotypic information was obtained by direct clinical evaluation and thorough chart review. Only families with phenotypes likely consistent with autosomal recessive etiology were included either based on the phenotype or the family history (Fig. 1). To ensure high molecular weight (HMW) gDNA, lymphoblastoid cell lines (LCL) were established from at least one patient except in Family F8602 where LCL were established from the parents of the deceased child for duo analysis. LCL were also used as an RNA source to confirm the impact of non-coding variants using RT-PCR and RT-qPCR as appropriate. Some families also consented to skin biopsies for the establishment of primary fibroblast cell lines, which were also used as a source of RNA. Additional consent was obtained for the publication of identifiable clinical images.

### Reanalysis of negative exomes

At least one clinical exome based on short-read sequencing with negative results was available for the index patient in each of the study cohort. The raw data were obtained and reanalyzed to consider a predetermined set of potential causes of negative reporting as described before [9]. Briefly, these causes include challenges related to phenotype (e.g., novel allelic disorders), pedigree structure (e.g., imprinting disorders masquerading as autosomal recessive phenotypes), positional mapping (e.g., double recombination events abrogating candidate autozygous interval), gene (e.g., novel gene-disease assertion) and variant (e.g., complex compound inheritance).

### Autozygome analysis

Axiom SNP Array was used for genomewide genotyping following the manufacturer’s protocol. Given the highly consanguineous nature of our population, runs of homozygosity > 2 Mb were used as surrogates of autozygosity as described before [17].

### Chromosomal microarray

We analyzed all cases that remained negative after lrWGS with CMA to exclude SVs that may have been missed by the 10 × depth of lrWGS. We followed the same protocol described before [18].

### Long-read whole genome sequencing

The integrity of extracted HMW gDNA quality was assessed according to PacBio requirements with FEMTO Pulse (Agilent Technologies, Inc. P-0003–0817), Qubit dsDNA High Sensitivity (ThermoFisher Scientific Q33230), and Nanodrop (ThermoFisher Scientific ND-8000-GL). The gDNA was sheared with Megaruptor3 (Diagenode, Denville, USA B06010003), and SMRTbell libraries were prepared using SMRTbell prep kit 3.0 (Pacific Biosciences of California, Inc 102–182-700) with PippinHT System (Sage Science HTP0001) size selection. Finally, sequencing libraries were set up with Binding Kit 3.2 (102–333-300) according to conditions specified in SMRTlink and sequenced with Sequel II Sequencing Kit 2.0 (101–820-200), SMRT cell 8 M Tray (101–389-001), and run for 30 h movie time with the recommended pre-extension time in adaptive loading mode on the Sequel IIe system.

To calculate the required read depth for detecting variants, we first sequenced one sample (16DG0856) to a 40 × depth on four SMRT cells. A subsampling technique was used to compare variant detection using 25%, 50%, 75%, and 100% of the reads, corresponding to 10 × , 20 × , 30 × , and 40 × coverage, respectively. As shown in Additional file 1: Fig. S2, we found that at 10 × coverage, about 3–12% fewer variants were detected compared to using the full set (i.e., 40 ×) of the reads, offering an acceptable compromise on cost efficiency. Therefore, the remaining samples were sequenced on one SMRT cell that yielded an average depth of 10 × . HiFi reads in BAM format were processed using the PacBio Human WGS Workflow (commit version: https://github.com/PacificBiosciences/pb-human-wgs-workflow-snakemake/commit/5045b4ecaf151b069ccb5421d4ab14ed34ffceb5). The workflow first aligns the reads from each SMRT cell to the reference genome (hg19) using *pbmm2* v2.17; next, the data were processed to detect small variants and structural variants using *DeepVariant* v1.3 and *pbsv* v2.8, respectively. Finally, the variants reported here were manually investigated together with the supporting reads using IGV plots to rule out potential technical errors.

### Candidate variant prioritization

We have followed our previously described pipeline of prioritizing candidate variants from exome sequencing [19]. Briefly, we prioritized novel (not previously published in literature or public databases including ClinVar) and very rare (MAF < 0.001) homozygous variants within the candidate autozygome of the index individual. Genes with established OMIM phenotypes were investigated first and, when negative, candidate genes were also considered by following the general framework put forth by ClinGen [20]. Variants were investigated by Sanger sequencing to confirm compatible segregation and RT-PCR was pursued where indicated using LCL- or fibroblast-derived RNA. In order to maximize the yield of lrWGS for classes that are missed by exome, we specifically reanalyzed all eligible cases for interpretation challenges prior to their inclusion in lrWGS (see above).

### Cloning-free reporter assay

Two transcriptional reporter constructs containing wild-type or mutant sequences of the *SLC4A4* promoter region were produced by the cloning-free reporter generation method as previously described in [21] and briefly outlined below. First, the following sites (promoter/5′UTR) were utilized to create transcriptional promoter constructs: WT: CAGCCTCCAACCCCGGCGGCGCGC or Mut: CCAGCCTCCAACCCTGGCGGCGCGC. Two different strategies were performed: a transcriptional reporter construct in which the entire sequence is included in the promoter or with CAGCCTCCAACCCCGGC-5′ CAGCCTCCAACCCCGGT-5′ which ends at the 5´start site.

The pCMV-RBGT1-SGFP was constructed as previously described [21]. The PCR products were generated directly from the pCMV-RBGT1-SGFP using: a forward primer that targets the vector sequence upstream of the 5´UTR/ coding region of SGFP and contains the desired wild-type and mutant sequences (Additional file 1: Table S1) and a reverse primer that is complementary to a downstream region of the 3′UTR. The HPLC-purified oligonucleotide primers were custom-synthesized by Integrated DNA Technologies (IDT). Two-step PCR approach was performed using the following reagents and conditions: 2.5 U HotStart Taq (Qiagen) and 0.2 U Pfx polymerase (Invitrogen, Carlsbad, CA) mix, 2 μl (100–200 ng) of the vector template, 1 × PCR buffer, 0.2 mM dNTPs, 0.2 μM primers, with the following cycle conditions: 95 °C for 10 min, 10 cycles of 94 °C, 10 min., 60 °C, 1 min., 72 °C, 2 min., followed by another 25 cycles of 94 °C for 1 min, 70 °C, 2:30 min., and final extension at 72 °C for 10 min. The PCR products were purified using Qiagen PCR purification columns to eliminate small PCR products, primers, and buffer enzymes. The PCR products were finally eluted in sterile water. The PCR products were run on a 1.2% agarose gel and visualized by ethidium bromide under UV light to verify size and quality.

HEK293 cell line was used for transient transfection of the reporter constructs. The cell line was obtained from the American Type Culture Collection (ATCC; Rockville, MD) and was propagated in MEM medium with 10% FBS and antibiotics at standard culture conditions (37 °C, 5% CO2). The cells (5 × 10^4^ cells per well in 96-well clear-bottom black plates (Matrix Technologies, Hudson, NH)) were transfected with 50 ng of purified constructs (expression-ready PCR products). Transfections were performed in a serum-free medium using Lipofectinamine 2000 (Invitrogen). All transfections were performed in several replicates. The pRPS30-RPF plasmid [22] was used at 10 ng for co-transfection to monitor transfection normalization. Fluorescence intensity was measured after 24 and 48 h, respectively. Pictures were taken using the EVOS high-performance fluorescence microscope (Thermo Fisher, USA). Exposure intensity and duration, gain, and other settings were kept constant to allow equal comparison of experiments. Fluorescence intensity was calculated using ImageJ software for processing and analyzing images. Data are presented as the mean ± SEM of total fluorescence intensity in each well, with replicate readings.

### Cell viability assay

Equal numbers of fibroblast cells from the affected individual (14DG1582) and two controls were seeded in a 6-well plate and allowed to adhere overnight. The cells were then treated with increasing concentrations of 5-fluorouracil dissolved in DMSO (0.01, 0.5, 0.1, 1, and 10 µM) for 24 h. Cells were then washed and stained with crystal violet blue to assess cell viability.

### Nuclear morphology assay

Equal numbers of fibroblast cells from the affected individuals (17DG0936 and 17DG0937) and two controls were seeded on microscope slides and allowed to adhere overnight. Cells were then stained with DAPI and imaged using Zeiss Imager.Z2. Envelope vs Surface Ratio (ESR) was measured using ImageJ as described in [23].

### Transmission electron microscopy

Fibroblast cells from patient (14DG2098) and control as well as LCL from patients (14DG2102, 14DG2107, and 17DG0429) and controls were fixed in 3% glutaraldehyde and processed by the Electron Microscopy core Research Facilities laboratory in the University of Utah using Leica Ultramicrotome UCT. Cell imaging was performed using JEOL JEM-1400.

## Results

Our cohort comprises 34 families in which a presumably autosomal recessive disease defied molecular diagnosis by clinical exome sequencing (short-read sequencing-based) and reanalysis performed on the index individual for each family (Fig. 1). The index patient in each family was subjected to an average of 10 × depth lrWGS except for Family F8602 where the low-quality DNA from the deceased index prompted us to proceed with duo lrWGS on both parents (Additional file 1: Table S2). Using autozygome-guided analysis of the lrWGS data, candidate variants were identified in 13 of the 34 families (38%). Additional file 1: Table S3 describes all the variants found within ROHs which were subsequently excluded.

### Long-read whole genome sequencing reveals molecular diagnoses not detectable by exome sequencing

**Fig. 2:**
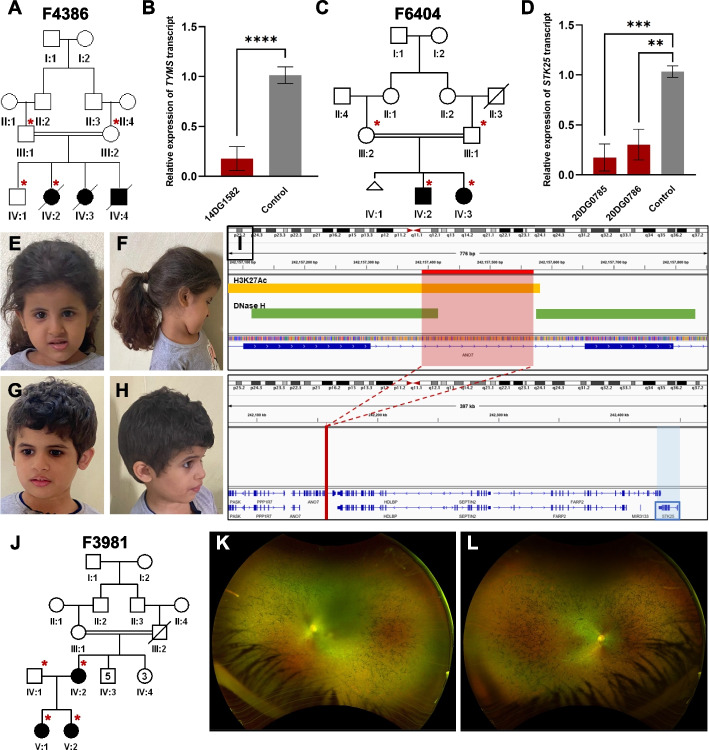
Structural variants that are not detected by exome. **A** Pedigree of family F4386 with three children who died as neonates with lactic acidosis. **B** RT-qPCR results showing reduced transcript levels of *TYMS* in one of the affected individuals compared to two independent controls. **C** Pedigree of family F6404 with two siblings affected with microcephaly, and developmental delay. **D** RT-qPCR data showing reduced expression of *STK25* in the two affected individuals compared to two independent control samples. **E**–**H** clinical images of the two siblings highlighting the microcephaly and lack of gross facial dysmorphism. **E** and **F** Clinical images of 20DG0785. **G** and **H** Clinical images of 20DG0786. **I** Genomic representation of the deletion with H3K27Ac and DNase hypersensitivity signals indicated. **J** Pedigree of family F3981 with two affected sisters with Leber congenital amaurosis and their affected mother with retinitis pigmentosa. **K** and **L** Widefield retinal imaging of the left and right retina of the mother (IV:2) showing rod cone dystrophy. Error bars denote standard deviation of at least 3 experiments. ****, ***, ** denote *p*-values < 0.0001, < 0.001, and < 0.01, respectively, using unpaired Student’s *t*-test

*1- TYMS* as a novel candidate gene for lethal neonatal lactic acidosis in family F4386:

14DG1582 is one of three neonates from the same family who died in the neonatal period with severe biochemically confirmed lactic acidosis and suspected mitochondrial dysfunction (Table 1 and Additional file 1: Table S4). Autozygome-guided lrWGS analysis revealed a homozygous structural variant (SV) (insertion of 270 bp in intron 3) in *TYMS* (hg19 Chr18:666999ins[270 bp]). *TYMS* (MIM 188350) encodes thymidylate synthase, a mitochondrial protein involved in the de novo and salvage dTTP pathways, deficiency of which leads to uracil misincorporation and mitochondrial dysfunction [24, 25]. RT-qPCR revealed a severe and significant reduction in *TYMS* expression in patient cells compared to controls (Fig. 2A and B). The variant was confirmed to be homozygous in one affected and heterozygous in the parents who did not have any evidence of dyskeratosis congenita, a condition that has recently been linked to digenic inheritance involving *TYMS* [26]. Similar to what has been reported in [26], patient-derived fibroblast cells are hypersensitive to 5-fluorouracil (5-FU), a known specific inhibitor of TYMS, compared to controls (Additional file 1: Fig. S1A).

**Table 1:** List of families with candidate variants not found in exome

Pedigree ID	# Affected members	Gender	Age at recruitment	Case ID	lrWGS Dx	Parental Consanguinity	Gene	Variant	Zygosity
F4386	3	M	16 days	14DG1582	*TYMS*-related lactic acidosis	First cousins	*TYMS*	NM_001071.4:c.455-2073ins of 270 bp	Homozygous
F6404	2	F	2 weeks	20DG0785	*ANO7* and *STK25*-related neurodevelopmental disorder	First cousins	*STK25*	NM_001370694.2:c.2178 + 83del of 184 bp (in *ANO7*)	Homozygous
F3981	3	F	5 months	17DG1097	Retinitis pigmentosa 88	Non-consanguineous	*RP1L1*	NM_178857.6:c.4026_4027insACAGA AGAAGGGCTGCAAGAAGAGGGGGTGC AGTTAGAGGAAACTAAAACAGAAGAAG GGCTGCAAGAAGAGGGGGTGCAGTTA GAGGAAACTAAAACAGAAGAAGGGCT GCAAGAAGAGGGGGTGCAGTTAGAGG GGACTAAA;p.Glu1343delinsThrGluGlu GlyLeuGlnGluGluGlyValGlnLeuGluGlu ThrLysThrGluGluGlyLeuGlnGluGluGlyV alGlnLeuGluGluThrLysThrGluGluGlyLe uGlnGluGluGlyValGlnLeuGluGlyThrLys Glu and NM_178857.6:c.3970_3971insGGACT AAAGTAATAGAAGGGCTGCAAGAAGA GAGGGTGCAGTTAGAGG;p.Glu1324del insGlyThrLysValIleGluGlyLeuGlnGluGl uArgValGlnLeuGluGlu	Compound heterozygous
F7974	2	F	10 years	20DG0235	*SLC4A4*-related band keratopathy	First cousins	*SLC4A4*	NM_001134742.2:c.-145C > T	Homozygous
F4591	4	M	2 years	14DG2098	*SNAP91*-related microcephalic primordial dwarfism	First cousins	*SNAP91*	NM_001242792.1:c.766-4799T > C	Homozygous
F5927	4	F	7 months	17DG0832	*LEMD2*-related neurodevelopmental disorder	Same tribe	*LEMD2*	NM_181336.4:c.1011-469_1011- 450del	Homozygous

2- Deletion of *STK25* regulatory region in a neurodevelopmental disorder (NDD) in family F6404:

20DG0785, a 3.5-year girl, and her 7-year-old brother (20DG0786) shared an apparently novel syndromic association of intellectual disability, microcephaly, and hearing loss (Table 1, Fig. 2C–H and Additional file 1: Table S4). Autozygome-guided lrWGS analysis revealed a homozygous 184 bp deletion SV (hg19 Chr2:242,157,389–242,157,573) in intron 20 of *ANO7*. The deletion spans a strong peak for H3K27Ac enhancer mark, enriched for TF binding, and is DNAse hypersensitive. This deletion is within the region of 2q37 microdeletion syndrome characterized by microcephaly and intellectual disability, where *STK25* was proposed to be a major contributor [27]. *STK25* (MIM 602255) is a germinal center kinase III (GCK III) that plays a role in serine-threonine liver kinase B1 (LKB1) signaling pathway. Its established role in neuronal and brain cortical development stems from its function in regulating neuronal polarization and morphology of the Golgi apparatus [28, 29]. We hypothesized that the deletion may impact *STK25* expression given its close proximity (276,549 kb away from the deletion). RT-qPCR data indeed confirmed a dramatic reduction of *STK25* expression in both siblings compared to controls (Fig. 2D and I). The deletion is predicted by the JASPER database [30] to impact the binding of multiple transcription factors, which are summarized in Additional file 1: Table S5.

3- Complex inheritance of SVs in *RP1L1* in family F3981:

F3981 consists of a mother (14DG0261) with late-onset retinitis pigmentosa and two daughters (17DG1097 and 14DG0524) with Leber congenital amaurosis (LCA). lrWGS in 17DG1097 (her sister 14DG0524 was confirmed to have the same finding) identified compound heterozygosity for two in-frame SVs in *RP1L1* (MIM 608581)*.* One SV was inherited from the mother who is homozygote for this variant (NM_178857.6:c.4026_4027insACAGAAGAAGGGCTGCAAGAAGAGGGGGTGCAGTTAGAGGAAACTAAAACAGAAGAAGGGCTGCAAGAAGAGGGGGTGCAGTTAGAGGAAACTAAAACAGAAGAAGGGCTGCAAGAAGAGGGGGTGCAGTTAGAGGGGACTAAA:p.Glu1343delinsThrGluGluGlyLeuGlnGluGluGlyValGlnLeuGluGluThrLysThrGluGluGlyLeuGlnGluGluGlyValGlnLeuGluGluThrLysThrGluGluGlyLeuGlnGluGluGlyValGlnLeuGluGlyThrLysGlu), while the other (NM_178857.6:c.3970_3971insGGACTAAAGTAATAGAAGGGCTGCAAGAAGAGAGGGTGCAGTTAGAGG:p.Glu1324delinsGlyThrLysValIleGluGlyLeuGlnGluGluArgValGlnLeuGluGlu) was inherited from the healthy heterozygous father (Table 1, Additional file 1: Table S4, and Fig. 2J–L).

**Fig. 3:**
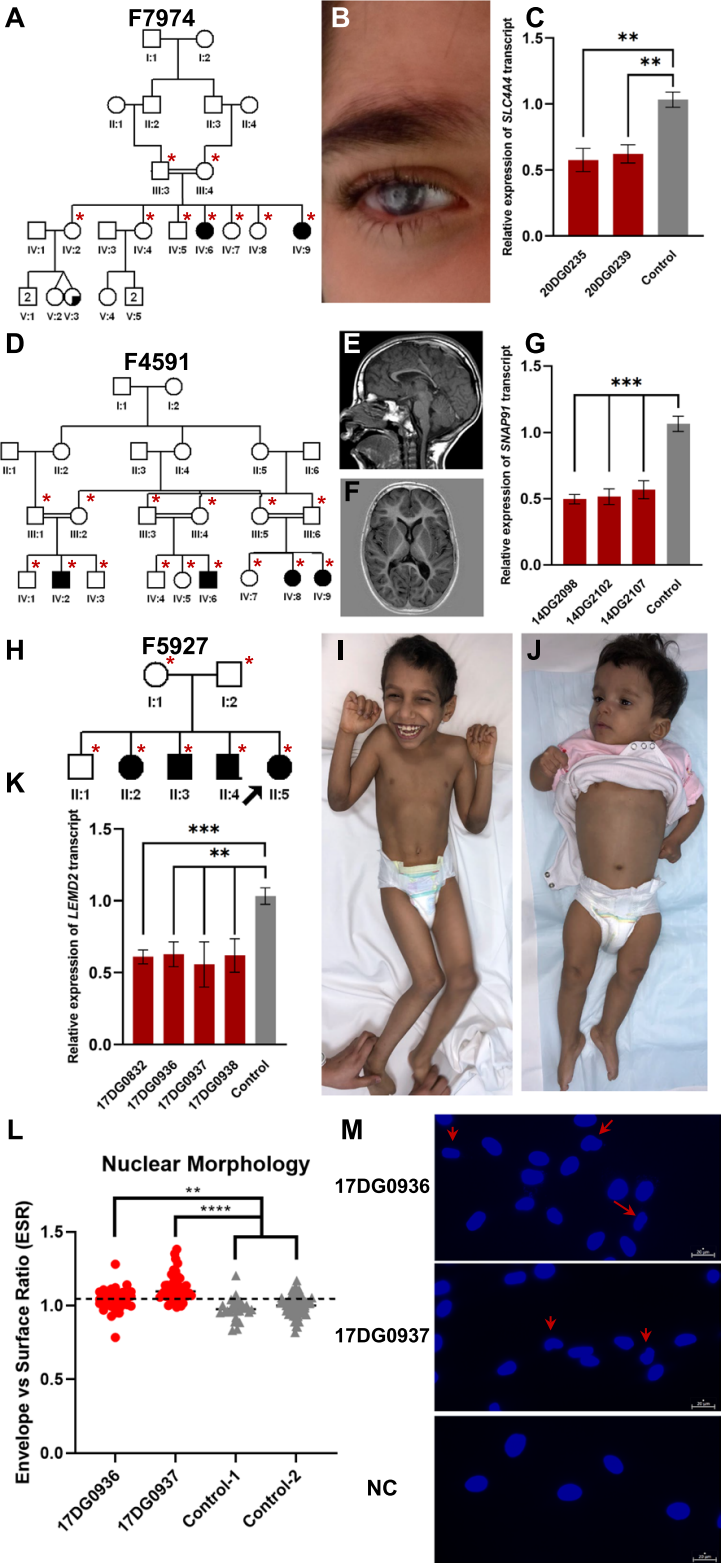
Challenging variants that are not detected by exome. **A** Pedigree of family F7974 with two sisters affected with band keratopathy. **B** Clinical images of individual (IV:6) highlighting keratopathy phenotype. **C** RT-qPCR experiment showing reduced *SLC4A4* transcript levels compared to two independent controls. **D** Pedigree of family F4591 with three cousins affected with microcephalic NDD. **E** and **F** MRI imaging of individual (IV:8) showing mild brain atrophy and thin corpus callosum. **G** RT-qPCR results demonstrating reduced expression of *SNAP91* in three patients compared to two independent controls. **H** Pedigree of family F5927 with four children affected with NDD. **I** and **J** Clinical images of individuals (II:3 and II:4) highlighting microcephaly and progressive spasticity. **K** RT-qPCR experiment showing consistently reduced *LEMD2* expression levels in samples from four affected siblings compared to two independent controls. **L** and **M** Nuclear morphology of patient cells compared to two independent controls and representative images highlighting abnormal nuclear morphology using red arrows. Error bars denote standard deviation of at least 3 experiments. ****, ***, ** denote *p*-values < 0.0001, < 0.001, and < 0.01, respectively, using unpaired Student’s *t*-test

4- A novel regulatory element variant in familial band keratopathy in family F7974:

20DG0235 and 20DG0239 are two siblings with non-syndromic band keratopathy (Table 1, Fig. 3A and B and Additional file 1: Table S4). Autozygome-guided lrWGS analysis revealed a novel homozygous SNV immediately upstream of the 5′UTR of *SLC4A4* (NM_001134742.2:c.-145C > T). *SLC4A4* (MIM 603345) recessive variants are known to cause band keratopathy as part of a multisystem syndrome, which these two siblings lack. We hypothesized that this may represent an instance of variable expressivity caused by a specific transcriptional dysregulation [31]. Indeed, RT-qPCR data revealed a significant reduction of *SLC4A4* expression in the two siblings compared to controls (Fig. 3C). To confirm the regulatory nature of the variant, a reporter assay was designed using WT and mutant sequences. Indeed, the mutant sequence showed 45–50% reduction in transcriptional activity compared to the control (Additional file 1: Fig. S1B and C).

*5- SNAP91* as a candidate gene for a novel NDD in family F4591:

14DG2098, 14DG2102, 14DG2107, and 17DG0429 are four relatives who share a severe NDD in the form of microcephaly, global developmental delay, spasticity, short stature, dysmorphic facies, and brain atrophy (Table 1, Fig. 3D–F, and Additional file 1: Table S4). They mapped to a single locus on Chr6:80,016,660–86,734,460 within which no candidate variants were identified by exome sequencing. However, lrWGS revealed a deep intronic homozygous SNV in *SNAP91* (NM_001242792.1:c.766-4799T > C) that was associated with a significant reduction in transcript level of the gene compared to control by RT-qPCR (Fig. 3G). *SNAP91* (MIM 607923) encodes synaptosomal-associated protein 91 which mediates endocytosis of synaptic vesicles (SVs). *Snap91*^−/−^ mice have a compatible phenotype in the form of growth retardation, spasticity, altered behavior, impaired neurotransmission, epileptic seizures, and premature death [32]. To test the effect of *SNAP91* reduction on the number of vesicles, we performed transmission electron microscopy on patient-derived lymphoblastoid and fibroblast cell lines. Data show that the patient cells had fewer number of vesicles compared to control cells (Additional file 1: Fig. S1D and E).

6- A novel transcript deleterious variant in *LEMD2* in family F5927:

17DG0832, 17DG0936, 17DG0937, and 17DG0938 are four siblings that share a phenotype comprising microcephaly, distal arthrogryposis, global developmental delay, failure to thrive, and diffuse white matter abnormalities (Table 1, Fig. 3H–J, and Additional file 1: Table S4). Autozygome analysis revealed a single candidate locus (chr6:21249760–35107170) within which lrWGS revealed a novel homozygous deletion (chr6:33,746,614–33,746,633del) in intron 4 of *LEMD2* (MIM 616312). RT-qPCR showed reduced expression of *LEMD2* transcript in all four affected siblings compared to control (Fig. 3K). *LEMD2* encodes a Lamin-related nuclear envelope protein and has been associated with autosomal dominant Marbach-Rustad progeroid syndrome based on two patients [23]. We propose this variant causes a novel autosomal recessive allelic disorder. To investigate the link between the *LEMD2* variant and the patients’ phenotype, we measured Envelope vs Surface Ratio as a proxy for nuclear morphology as previously described in [23]. Indeed, patient-derived fibroblasts had a dysmorphic nuclear morphology compared to controls (Fig. 3L and M).

### Long-read whole genome sequencing highlights interpretation challenges by exome

**Fig. 4:**
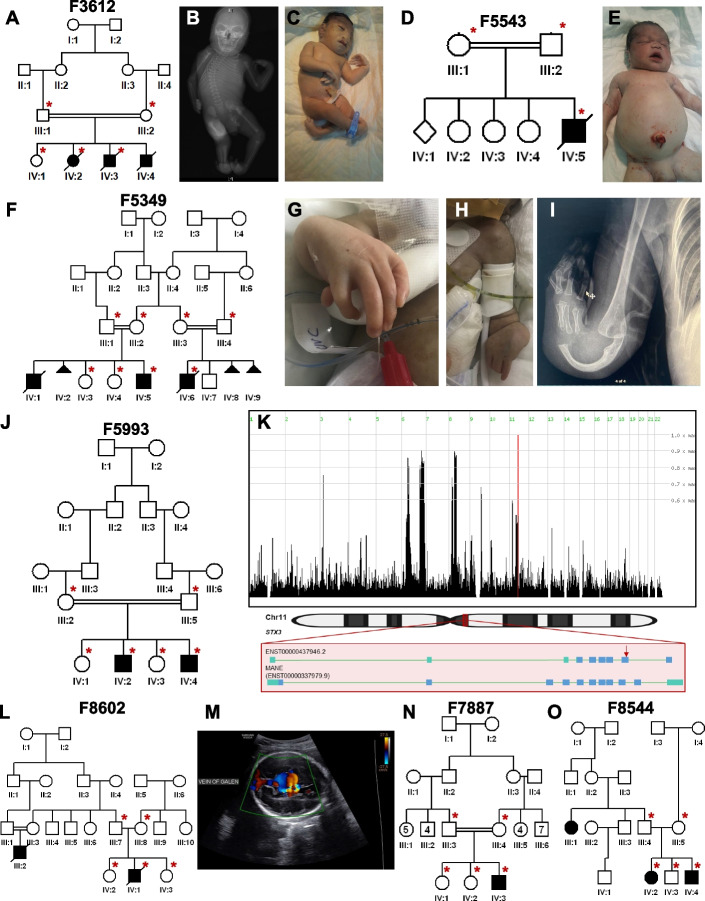
Families solved with variants that represented interpretation challenges. **A** Pedigree of family F3612 with three stillbirths all presenting with microcephaly and skeletal dysplasia phenotypes. **B** and **C** X-ray and clinical image of an affected individual (IV:4) highlighting radial ray deficiency. **D** Pedigree of family F5543 with an affected baby with polycystic kidney disease. **E** Clinical image of individual (IV:5) with Potter facies and swollen abdomen. F) Pedigree of family F5349 referred to us with two affected cousins both presenting with severe limb malformations. **G** and **H** Clinical images of affected individual (IV:6) with absent radius and hypoplasia of the ulna. **I** X-ray images of the right hand of affected individual (IV:6) highlighting absent radial ray and bowed ulna. **J** Pedigree of family F5993 with two brothers affected with retinal dystrophy. **K** (top) Genomic representation of identical haplotypes between two families homozygous for the same *STX3* variant. HomozygosityMapper shows that they map to a single locus on Chr.13; (bottom) schematic representation of two *STX3* transcripts, one where the variant is a frameshift insertion and the other (MANE select) as deep intronic. **L** Pedigree of family F8602 with two cousins affected with vein of Galen malformation. **M** Brain ultrasound imaging of individual (IV:2) showing dilatation of the veins of Galen. **N** Pedigree of family F7887 with NDD. **O** Pedigree of family F8544 with two siblings and a cousin affected with NDD. Illustrations at the bottom of panel (**K**) were created using BioRender.com

- 1- A Diamond-Blackfan syndrome-like phenotype caused by *FLVCR1* deficiency in family F3612:

13DG1395 and 16DG0856 are two of three stillborn babies with microcephaly, intrauterine growth retardation, severe craniofacial dysmorphism with clefting and severe skeletal malformations in a pattern highly consistent with Diamond-Blackfan syndrome (Table 2, Fig. 4A–C and Additional file 1: Table S4). Autozygome-guided lrWGS analysis revealed a homozygous 4-bp deletion (NM_014053.4:c.1593 + 5_1593 + 8del) in *FLVCR1* (MIM 609144). This canonical splicing variant was also identified on exome sequencing but was dismissed as an incidental finding because it had been reported in a family with adult-onset ataxia and retinitis pigmentosa, the only listed phenotype in OMIM [33]. However, the mouse knockout of *Flvcr1* is known to phenocopy Diamond-Blackfan anemia [34]. Further investigation revealed that the previously reported family with our variant was in fact compound heterozygous for this variant in trans with a presumably milder missense variant (c.1547G > A:p.(Arg516Gln)). Thus, we propose that our family represents the true null phenotype of *FLVCR1* in humans. Indeed, this case will be part of a cohort of similar cases caused by severe biallelic variants in *FLVCR1* (Calame et al., manuscript in preparation). RT-PCR experiment showed that the variant causes a splicing defect (skipping of exon 9) and an early truncation of the protein (r.1526_1593del;p.(Ala509Aspfs*4)).

- 2- A high-frequency *PKHD1* variant causes lethal polycystic kidney disease in family F5543:

**Table 2:** List of families with candidate variants representing interpretation challenges

Pedigree ID	# Affected members	Gender	Age at recruitment	Case ID	lrWGS Dx	Parental consanguinity	Gene	Variant	Zygosity	Reason missed by WES
F3612	2	M	Stillbirth	16DG0856	-related Diamond-Blackfan anemia	First cousins	*FLVCR1*	NM_014053.4:c.1593 + 5_1593 + 8del	Homozygous	Novel allelic disorder
F5543	1	M	Neonate	16DG0518	Polycystic kidney disease 4, with or without hepatic disease	Second cousins	*PKHD1*	NM_138694.4:c.2180A > G;p.(Asn727Ser)	Homozygous	High MAF in the local population
F5349	2	M	1 month	20DG1379	Fanconi anemia, complementation group J	First cousins	*BRIP1*	NM_032043.3:c.2392C > T;p.(Arg798*)	Homozygous	Intrafamilial genetic heterogeneity
F5993	2	M	1 year	18DG0095	*STX3*-related retinal dystrophy	First cousins	*STX3*	NM_004177.5:c.786 + 190dup (ENST00000437946.2:c.455dup;p.(Asp152Glufs*11))	Homozygous	Isoform confusion
F8602	2	F	Neonate	21DG0165	*NID1*-related vein of Galen malformation	Same tribe	*NID1*	NM_002508.3:c.3394C > T;p.(Arg1132Trp)	Homozygous	Novel allelic disorder
F7887	1	M	3 years	20DG0198	*ABHD12*-related developmental regression	First cousins	*ABHD12*	NM_001042472.3:c.952G > A;p.(Val318Met)	Homozygous	Atypical presentation
F8544	2	F	14 years	20DG1533	*C1orf109*-related neurodevelopmental disease	Same tribe	*C1orf109*	NM_001350767.2:c.224G > C:p.(Arg75Pro)	Homozygous	Erroneous application of MAF filter

16DG0518 is a stillborn baby with severe bilateral polycystic kidney disease (Table 2, Fig. 4D and E, and Additional file 1: Table S4). Autozygome-guided lrWGS analysis revealed a homozygous SNV (NM_138694.4:c.2180A > G;p.(Asn727Ser)) in *PKHD1* (MIM 606702). This missense variant was also identified on exome sequencing but was dismissed because of its unusually high local frequency (MAF 0.006196). However, an updated local exome database of 13,473 exomes revealed that this variant was observed in the homozygous state in 2 children who died with an identical phenotype and in the shared heterozygous state in a consanguineous couple who lost children with lethal polycystic kidney disease. Haplotype analysis confirmed that this is indeed a previously unrecognized common founder variant in the local population.

- 3- Genetic heterogeneity of severe limb anomalies in a consanguineous family F5349:

20DG1379 is a 2.5-year child with radial ray deficiency as part of a syndromic presentation and has a similarly affected deceased brother and a cousin with severe limb malformations (Table 2, Fig. 4F–I, and Additional file 1: Table S4). Autozygome-guided lrWGS analysis in the nuclear family revealed a homozygous SNV (NM_032043.3:c.2392C > T;p.(Arg798*)) in *BRIP1* (MIM 605882). This variant was also identified on exome sequencing but was ignored because it did not reside within a shared autozygous interval with the affected cousin. Indeed, segregation analysis of this pathogenic variant, which fully explains the phenotype, showed that the cousin is not homozygous, which confirms the genetic heterogeneity of the phenotype in this family. Rather, the cousin with split hand and foot malformation (SHFM) was subsequently found to have on chromosomal microarray a homozygous deletion in 12p11.23 (27,293,748–27,796,425). We propose this novel homozygous deletion as a potential cause through a position effect as has been demonstrated in other SHFM loci.

- 4- Isoform confusion in *STX3*-related retinal dystrophy in family F5993:

18DG0095 and 18DG0094 are two siblings affected by non-syndromic retinal degeneration in the form of diffuse RPE changes (Table 2, Fig. 4J, and Additional file 1: Table S4). Autozygome-guided lrWGS analysis revealed a homozygous indel (NM_004177.5:c.786 + 190dup) in *STX3* (MIM 600876). Despite the deep intronic nature of this variant, it was also captured by exome sequencing but was ignored because in silico prediction and subsequent RT-PCR failed to show an abnormal impact on splicing. Further investigation of this variant revealed that it is exonic and truncating in one isoform that is only listed in Ensembl where the nomenclature is (ENST00000437946.2:c.455dup;p.(Asp152Glufs*11)). The same variant was subsequently identified in 2 unrelated patients with an identical phenotype, and haplotype analysis confirmed the founder nature of this variant (Fig. 4K). Interestingly, this is the first demonstration of non-syndromic retinal dystrophy linked to *STX3*, a gene that has thus far been only implicated in the syndrome of retinal dystrophy with microvillus inclusion [35]. Of note, upon investigating the expression of this transcript, it was found to be expressed mainly in the brain and testis as opposed to the canonical transcript, which is expressed in the brain but enriched in the small intestine and other tissues [36].

- 5- A novel allelic disorder attributed to *NID1* in family F8602:

21DG0165 is a neonate who died of high throughput heart failure as a complication of vein of Galen malformation (Table 2, Fig. 4L and M, and Additional file 1: Table S4). A cousin also died in the neonatal period of the same condition. Only low-quality DNA was recovered from the index child. Therefore, we performed lrWGS on both parents assuming the shared carrier status of an autosomal recessive variant. Autozygome-guided lrWGS analysis revealed that both parents shared a novel SNV NM_002508.3:c.3394C > T;p.(Arg1132Trp) in *NID1* (MIM 131390) with compelling in silico predictions (SIGMA + 0.65, CADD 24.1, PolyPhen 0.999, SIFT 0.001). NID1 is a member of the nidogen family of basement membrane glycoproteins, which play a role in cellular interactions with the extracellular matrix through interaction with several other components of basement membranes. Subsequent Sanger sequencing confirmed the homozygous status of the variant in the index child and absence in the unaffected siblings. To date, there is no phenotypic OMIM listing for *NID1-*related phenotype although we have previously published congenital stroke in two siblings who shared a novel homozygous splicing variant confirmed by RT-PCR [37]. Indeed, *Nid1*-/- mice were also noted to have abnormal motor control and this was tracked to the highly abnormal basement membrane of brain capillaries [38]. We propose that the arteriovenous malformation observed in the family we report here may be a novel allelic disorder linked to *NID1*. This novel phenotypic aspect precluded the identification of this variant on the exome despite being fully captured and called.

- 6- Atypical presentation in a case with *ABHD12* variant in family F7887:

20DG0198 is a 6-year-old child with developmental regression (Table 2, Fig. 4N, and Additional file 1: Table S4). Autozygome-guided lrWGS analysis revealed a novel homozygous SNV (NM_001042472.3:c.952G > A;p.(Val318Met)) in *ABHD12* (MIM 613599) with compelling in silico predictions (SIGMA + 0.78, CADD 28.3, PolyPhen 0.955, SIFT 0.003). Although this variant was also called by exome sequencing, it was dismissed because of the atypical presentation compared to OMIM phenotype (absence of hearing loss, retinitis pigmentosa, and cataract).

- *7- C1orf109* as a candidate gene for a novel NDD in family F8544:

20DG1533 and 20DG1534 are two siblings who shared a severe NDD in the form of progressive microcephaly, global developmental delay, and brain atrophy (Table 2, Fig. 4O, and Additional file 1: Table S4). Autozygome-guided lrWGS analysis revealed a homozygous SNV (NM_001350770.2:c.224G > C;p.(Arg75Pro)) in *C1orf109* (MIM 614799) with compelling in silico predictions (CADD 24.8, PolyPhen 0.987, SIFT 0.2). Although this variant was also called by exome sequencing, it was ignored because the analysis pipeline was erroneously set to exclude variants that are present in the homozygous state in the database. Subsequently, we identified the source of the error as a homozygous individual who shared the same phenotype. GeneMatcher submission revealed an ongoing study with a large cohort on *C1orf109*-related NDD (manuscript in preparation).

### The limitation of low-depth lrWGS

**Fig. 5:**
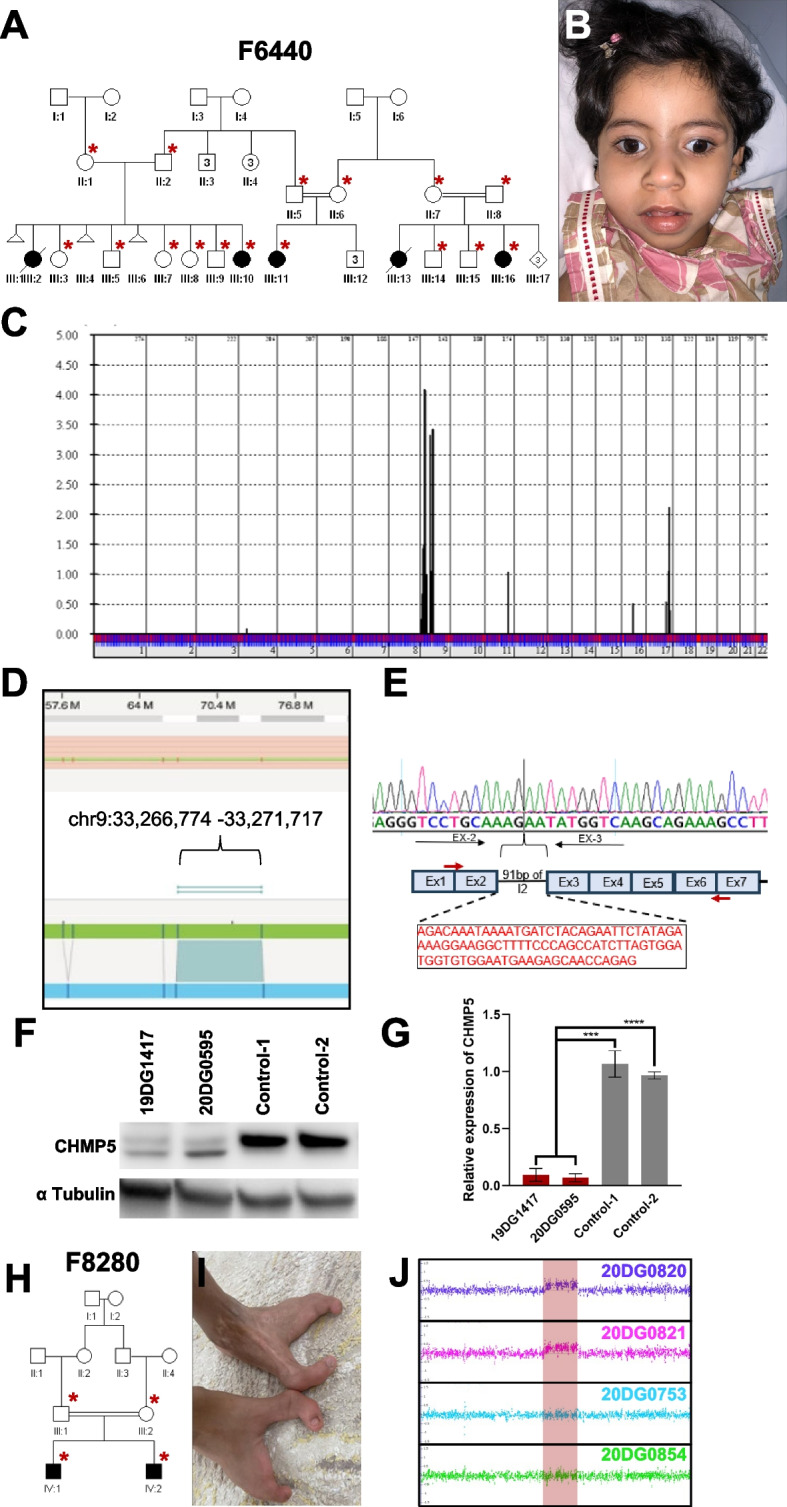
Variants that were not detected by lrWGS. **A** Pedigree of family F6440 with three stillbirths all presenting with profound global developmental delay, microcephaly, epilepsy, and scoliosis. **B** Clinical image of an affected individual (III:10) highlighting microcephaly. **C** EasyLinkage analysis showing a single locus at chr9:31537680–79306780 with LOD of 4. **D** Screenshot of Bionano analysis output identifying an insertion (chr9:33,266,774–33,271,717) disrupting *CHMP5*. **E** RT-PCR experiment showing 91 bp insertion in intron 2 (I2). **F** Western blot experiment showing reduction of CHMP5 protein levels in two affected individuals compared to two independent controls. Alpha-Tubulin was used as loading control. **G** Relative quantification of CHMP5 protein levels showing ~ 80% reduction compared to controls. **H** Pedigree of family F8280 with two siblings affected with SHFM. **I** Clinical image of limb malformation of affected individual (IV:1). **J** Chromosomal microarray output from family F8280 showing heterozygous duplication in the known SHFM locus that was absent in the parents

The above results show that despite the compromise on the depth of lrWGS to save cost, we were able to identify candidate variants in 38% of the study families with the help of positional mapping. Nonetheless, we were interested to explore the contribution of the low-depth strategy to the 62% negative families. We chose F6440 as the most compelling family for further analysis because the phenotype maps to a single locus in which lrWGS failed to identify any candidate variant. Family F6440 comprises multiple affected cousins, three of whom (19DG1417, 20DG0595, and 20DG0706) were available for testing (Fig. 5A). The shared phenotype comprises profound global developmental delay, microcephaly, central hypotonia, peripheral spasticity, epilepsy, and scoliosis (Fig. 5B). Brain imaging showed white matter disease suggesting leukodystrophy. Linkage analysis revealed a single locus shared between the three affected cousins (chr9:31,537,680–79306780) with LOD of 4 (Fig. 5C). We utilized Optical Genome Mapping to investigate the possible presence of structural variants missed by lrWGS. Indeed, we were able to identify a novel insertion (chr9:33,266,774–33,271,717) disrupting the reading frame of *CHMP5* (MIM 610900) (Fig. 5D–F). *CHMP5* is a compelling candidate gene for this novel NDD because null mice displayed embryonic lethality during organogenesis with abnormal neural plate and tube morphology [39].

Another case where lrWGS failed to identify the causal variant is family F8280 with two affected siblings 20DG0820 and 20DG0821 clinically diagnosed with SHFM (Fig. 5G and H). The normal clinical exam of the parents and their consanguineous nature suggested a potential autosomal recessive etiology. However, chromosomal microarray revealed a heterozygous duplication in the classical Split-hand/foot malformation 3 locus 10q24.31q24.32(102,950,203_103,472,860) × 3. The heterozygous duplication was observed in both affected siblings and absent in parents, indicating parental gonadal mosaicism.

## Discussion

The question of what should be done next when a genetic diagnosis is not made by exome sequencing is timely and has been the topic of intense discussion with no clear guidance [3, 40, 41]. In this study, the largest to date on the utility of lrWGS in patients with suspected autosomal recessive diseases, we explore the various factors that lead to negative exome sequencing and the role of lrWGS as a reflex test. We show that while lrWGS clearly uncovers causal variants that are missed by exome, interpretation challenges remain an important etiology of non-diagnostic exomes.

As expected, lrWGS demonstrated a clear advantage in detecting SVs, an important class of variants that remain challenging for short-read sequencing even with improved bioinformatic handling of the data. Nearly half of the identified candidate variants were SVs and SNVs that were missed by exome. It should be noted that the preselection of autosomal recessive phenotypes in our highly consanguineous cohort made it less likely for other classes of challenging variants to be identified, e.g., repeat expansion and chromosomal rearrangements, since these tend to be dominant in nature. What is surprising, however, is that the other half of the candidate variants we identified by lrWGS were similarly detected by exome sequencing and yet were not highlighted as likely candidates. This is despite the fact that all exome files were carefully reanalyzed for a long list of challenges that we identified based on a previously published study involving the systemic analysis of such challenges [9]. For example, despite the emphasis we placed on the phenomenon of complex compound inheritance where a given autosomal recessive variant can express phenotypically in distinct ways depending on which other variant exists *in trans*, we failed to invoke this phenomenon in Family F3612. This family, which harbors a previously reported *FLVCR1* pathogenic variant, showcases how this phenomenon can be very challenging to address even when specifically considered. The dramatic difference between an adult-onset ataxia-retinitis pigmentosa syndrome and an embryonic lethal major malformation syndrome makes it hard to consider the possibility that the same variant can be responsible for both conditions with the former being caused by compound heterozygosity with a mild variant and the latter by homozygosity for this null variant. We also note the allele frequency challenge observed in a *PKHD1* variant, the intrafamilial genetic heterogeneity for major limb malformations associated with a *BRIP1* variant, the isoform challenge associated with a *STX3* variant (first example of *STX3*-related non-syndromic retinal dystrophy) and the phenotypic challenge associated with *NID1* as a novel candidate cause of autosomal recessive vein of Galen malformation.

There are limitations in this study. Despite being the largest to date on autosomal recessive phenotypes, we note the need for much larger cohorts. In order to limit the cost of the study, we resorted to a limited average depth of 10 × . Thus, it is possible that families that remained negative may benefit from a higher depth of sequencing. Indeed, as shown in Family F6440 that mapped to a single locus, low-depth lrWGS failed to identify the likely causal variant within this locus, which was a novel large insertion that may have been identified by higher-depth sequencing. Similarly, the SHFM duplication on chr10q24.3 was missed in F8280. Although our study specifically targeted phenotypes that are likely to be autosomal recessive in etiology, which may limit the generalizability of the findings, this can also be viewed as an advantage because it allowed us to showcase the added value of positional mapping when interpreting lrWGS just as we have shown for srWGS [7]. Finally, we note that despite the compelling nature of the novel candidate genes revealed by our analysis, the proposed gene-disease assertions remain limited pending future cases. Each of these genes has been submitted to publicly available databases to facilitate gene matching.

## Conclusions

Our data clearly demonstrate the important role of non-coding DNA as well as the continued need to address the interpretation challenge in parallel with efforts to address the detection challenge by improving the sequencing technology itself. We show a number of novel gene-disease relations (novel candidate genes and novel phenotypes of established genes) that await confirmation by future cohorts. We propose a path forward for the implementation of lrWGS in the setting of autosomal recessive diseases in a way that maximizes its utility by exploiting the power of positional mapping.

### Supplementary Information

**Additional file 1: Fig. S1.** Functional validation of *TYMS*, *SNAP91*, and *SLC4A4* variants. **Fig. S2.** Comparing variant detection using different depths on the Pacific Bioscience Sequel IIe platform. **Fig. S3.** PacBio data describing variants highlighted in this study. **Table S1.** Sequences of primers used for the cloning-free reporter assay. **Table S2.** Coverage statistics for PacBio-sequenced samples. **Table S3.** List of variants identified within ROHs which were ultimately excluded. **Table S4.** Detailed listing of the study cohort. **Table S5.** List of transcription factors predicted to bind to the deleted region in F6404 with JASPER confidence scores.

## Data Availability

All data supporting the findings of this study are available either within the article, supplementary data files, or from the authors upon reasonable request. Requests can be directed to the corresponding author (falkuraya@kfshrc.edua) and a response should be expected within 30 days. The raw genome and exome sequence data are protected and are not available due to data privacy laws.
