# Supplementary material for: Beyond the exome: utility of long-read whole genome sequencing in exome-negative autosomal recessive diseases
Source: Genome Med. 2023 Dec 14;15:114. doi: 10.1186/s13073-023-01270-8 (PMC10720148; doi:10.1186/s13073-023-01270-8)
Supplement: Supplementary file 1 — Additional file 1: Fig. S1. Functional validation of TYMS, SNAP91, and SLC4A4 variants. Fig. S2. Comparing variant detection using different depths on the Pacific Bioscience Sequel IIe platform. Fig. S3. PacBio data describing variants highlighted in this study. Table S1. Sequences of primers used for the cloning-free reporter assay. Table S2. Coverage statistics for PacBio-sequenced samples. Table S3. List of variants identified within ROHs which were ultimately excluded. Table S4. Detailed listing of the study cohort. Table S5. List of transcription factors predicted to bind to the deleted region in F6404 with JASPER confidence scores. [file 13073_2023_1270_MOESM1_ESM.pdf]

Fig. S1

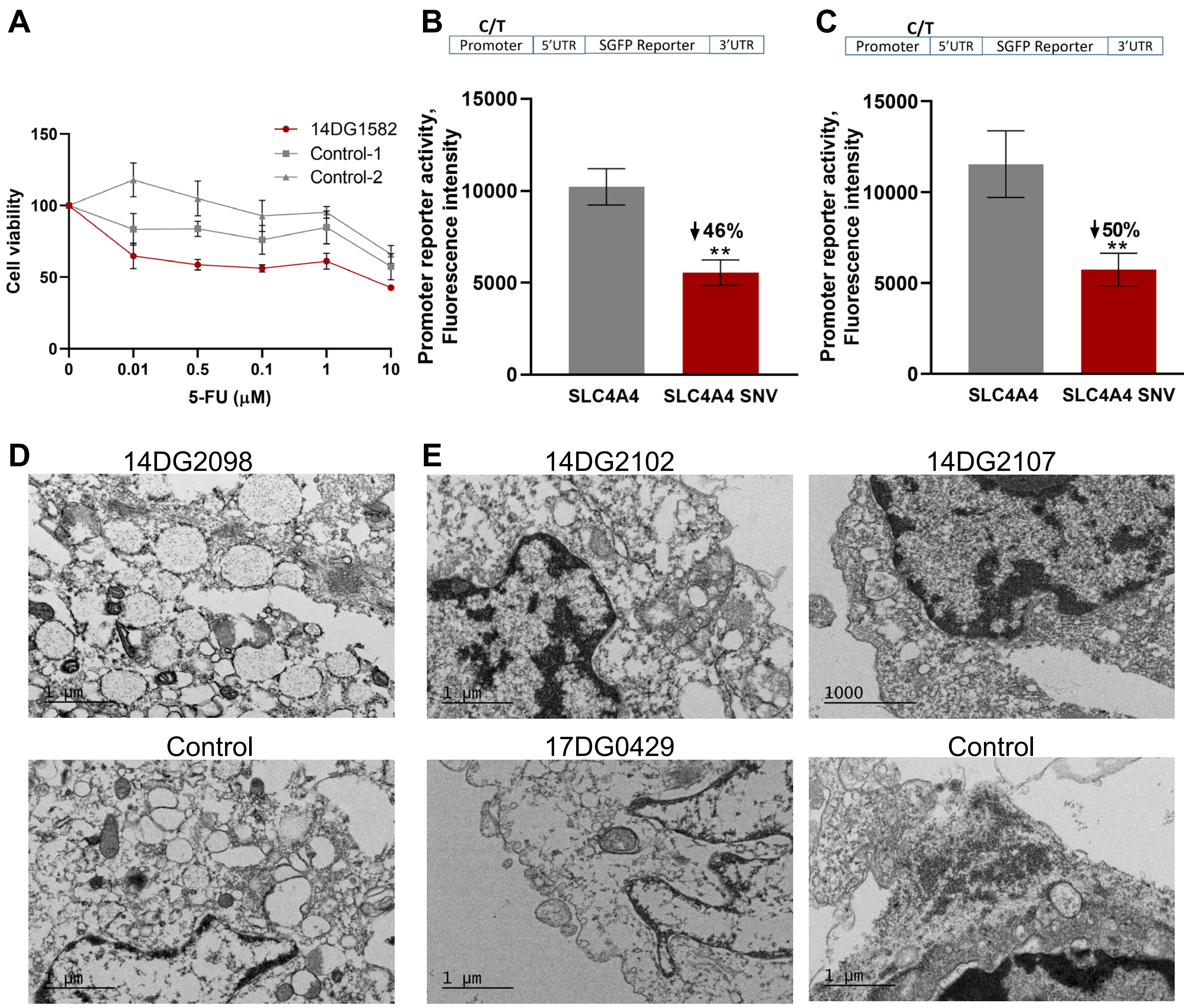

A
14DG1582
Control-1
Control-2
Cell viability
150
100
50
0
0
0.01
0.5
0.1
1
10
5-FU (μM)
B
C/T
Promoter
5'UTR
SGFP Reporter
3'UTR
Promoter reporter activity,
Fluorescence intensity
15000
10000
5000
0
46%
**
SLC4A4
SLC4A4 SNV
C
C/T
Promoter
5'UTR
SGFP Reporter
3'UTR
Promoter reporter activity,
Fluorescence intensity
15000
10000
5000
0
50%
**
SLC4A4
SLC4A4 SNV
D
14DG2098
1 μm
Control
1 μm
E
14DG2102
1 μm
14DG2107
1000
17DG0429
1 μm
Control
1 μm

Fig. S2

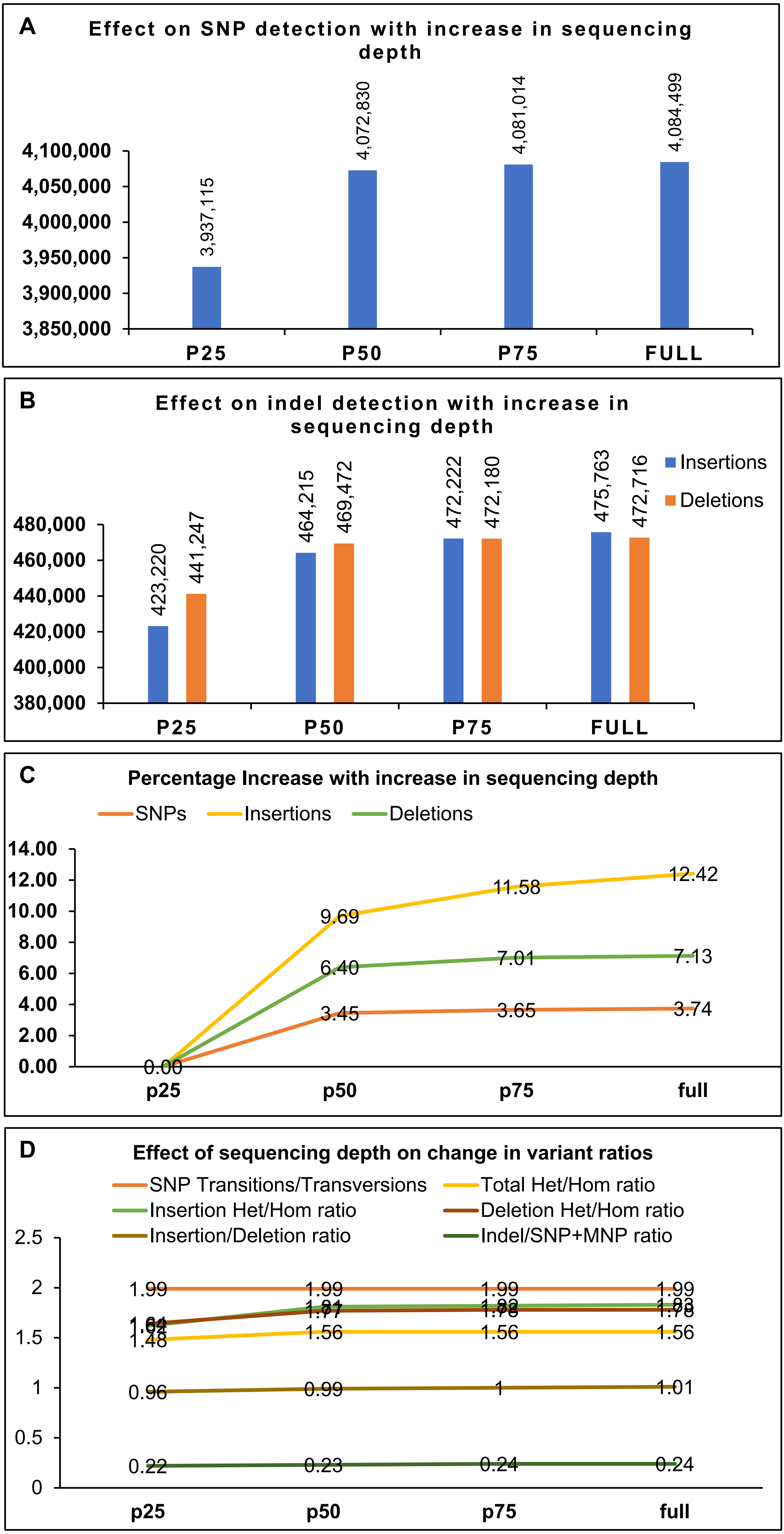
A
Effect on SNP detection with increase in sequencing depth
3,937,115
4,072,830
4,081,014
4,084,499
4,100,000
4,050,000
4,000,000
3,950,000
3,900,000
3,850,000
P25
P50
P75
FULL
B
Effect on indel detection with increase in sequencing depth
Insertions
Deletions
423,220
441,247
464,215
469,472
472,222
472,180
475,763
472,716
480,000
460,000
440,000
420,000
400,000
380,000
P25
P50
P75
FULL
C
Percentage Increase with increase in sequencing depth
SNPs
Insertions
Deletions
14.00
12.00
10.00
8.00
6.00
4.00
2.00
0.00
9.69
11.58
12.42
6.40
7.01
7.13
3.45
3.65
3.74
p25
p50
p75
full
D
Effect of sequencing depth on change in variant ratios
SNP Transitions/Transversions
Total Het/Hom ratio
Insertion Het/Hom ratio
Deletion Het/Hom ratio
Insertion/Deletion ratio
Indel/SNP+MNP ratio
2.5
2
1.5
1
0.5
0
1.99
1.48
1.56
0.96
0.99
1
1.01
0.22
0.23
0.24
p25
p50
p75
full

Fig. S3

A

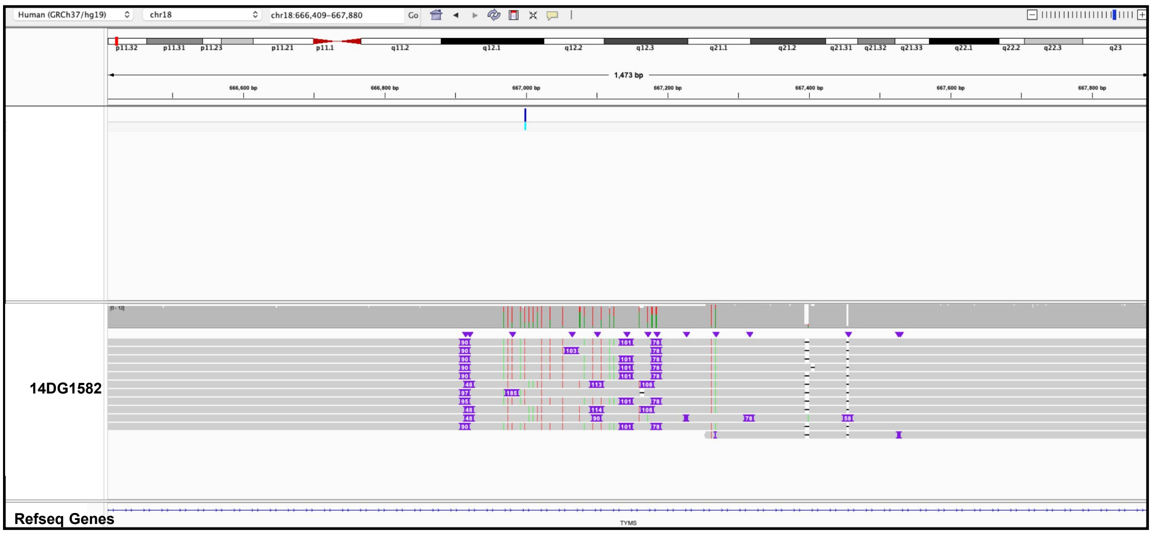

TYMS
NM_001071.4:c.455-2073ins of 270bp

B

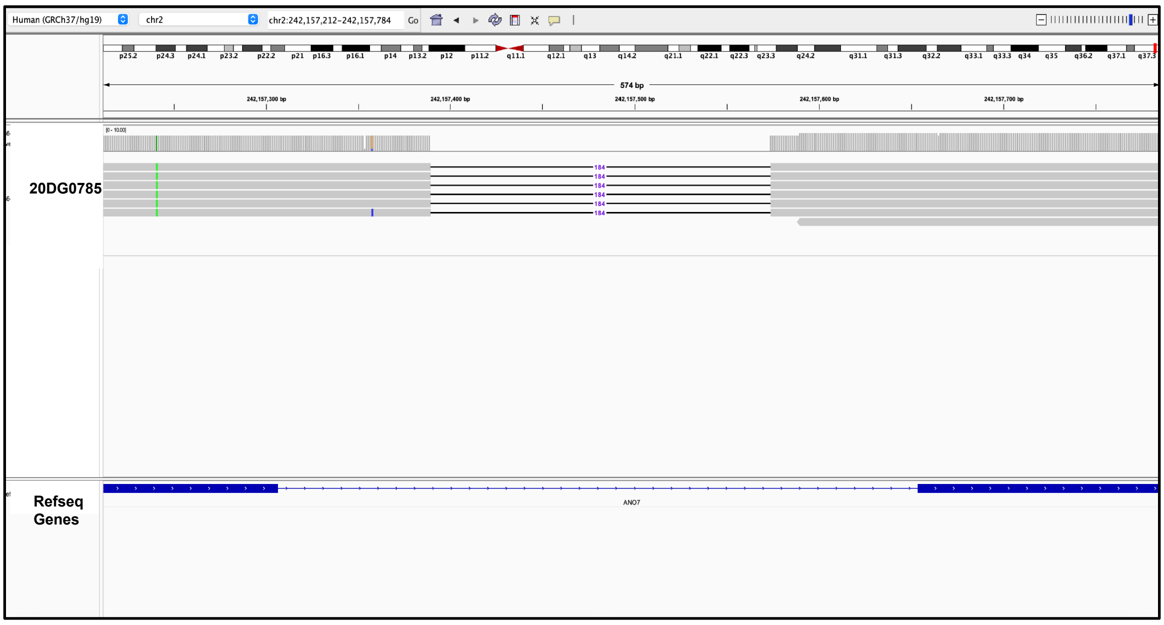

ANO7
NM_001370694.2:c.2178+83del of 184bp

C

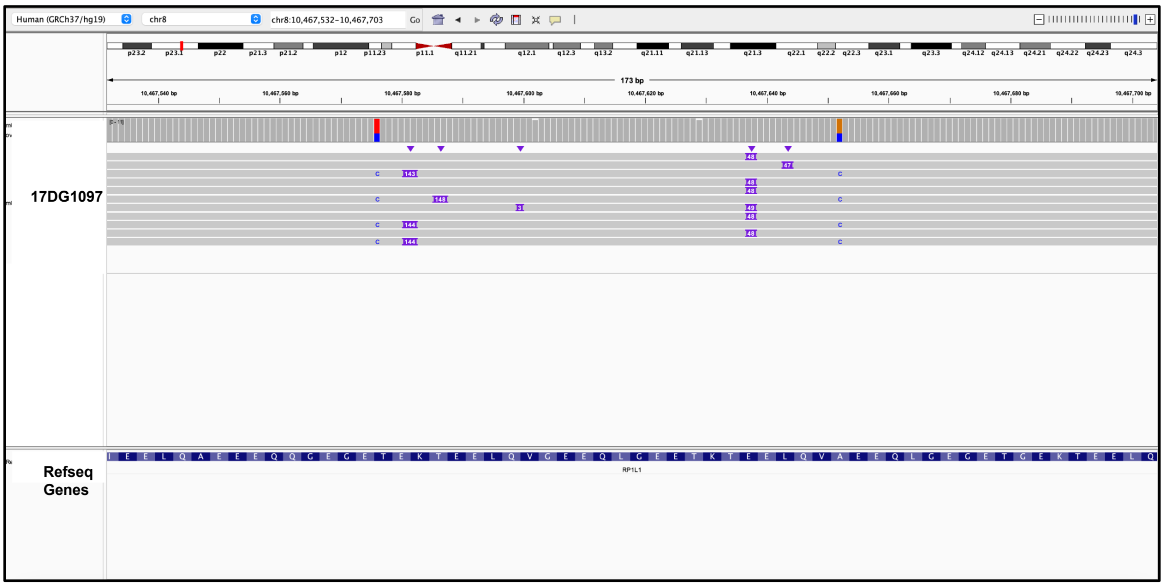

RP1L1
NM_178857.6:c.4026_4027InsACAGAAGAAGGGCTGCAAGAAGAGGGGGTGCAGTTAGAGGAAACTAAA ACAGAAGAAGGGCTGCAAGAAGAGGGGGTGCAGTTAGAGGAAACTAAAACAGAAGAAGGGCTGCAAG AAGAGGGGGTGCAGTTAGAGGGGACTAAA
and
NM_178857.6:c.3970_3971insGGACTAAAGTAATAGAAGGGCTGCAAGAAGAGAGGGTGCAGTTAGAGG

D

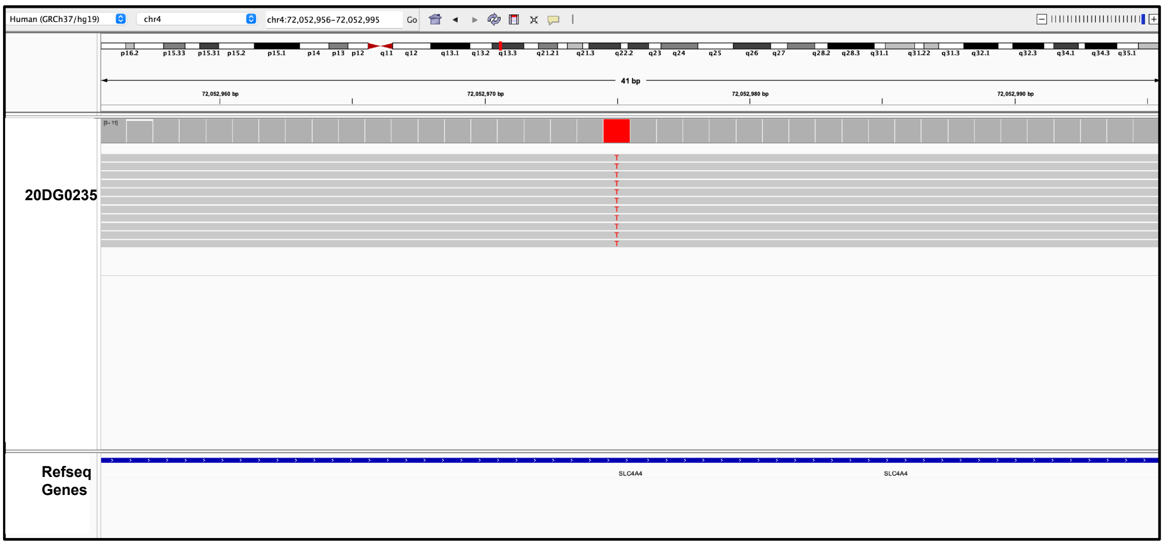

SLC4A4
NM_001134742.2:c.-145C>T

E

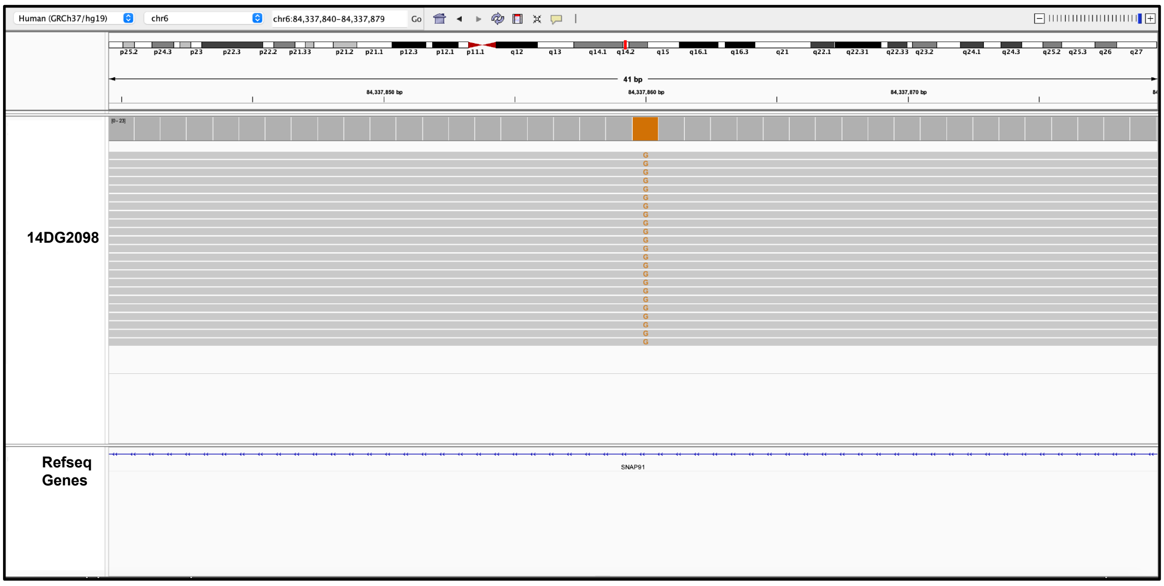

SNAP91
NM_001242792.1:c.766-4799T>C

F

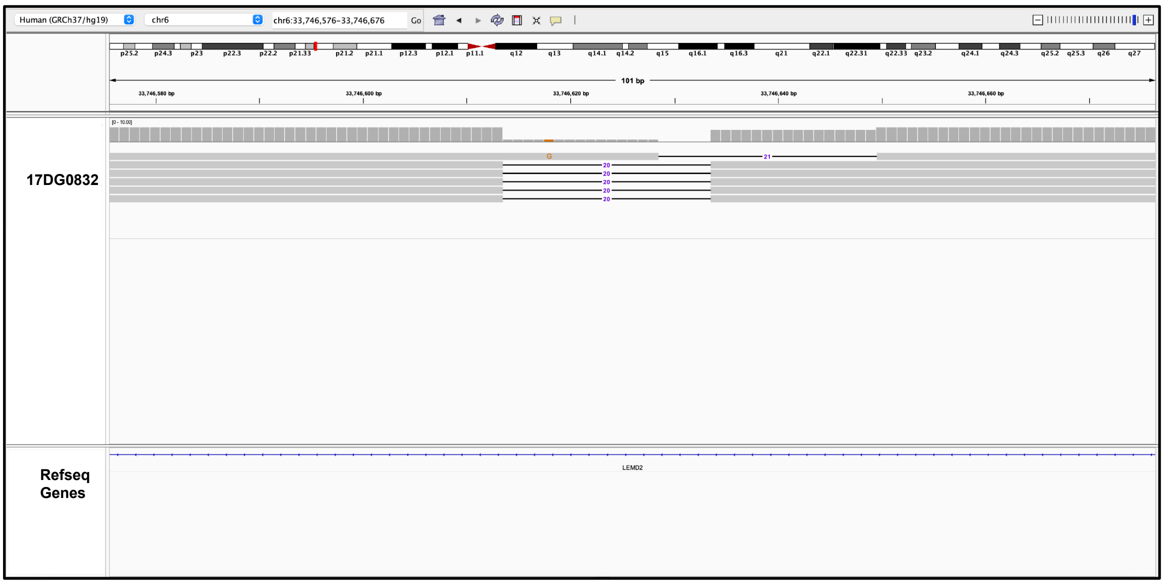

LEMD2
NM_181336.4:c.1011-469_1011-450del

G

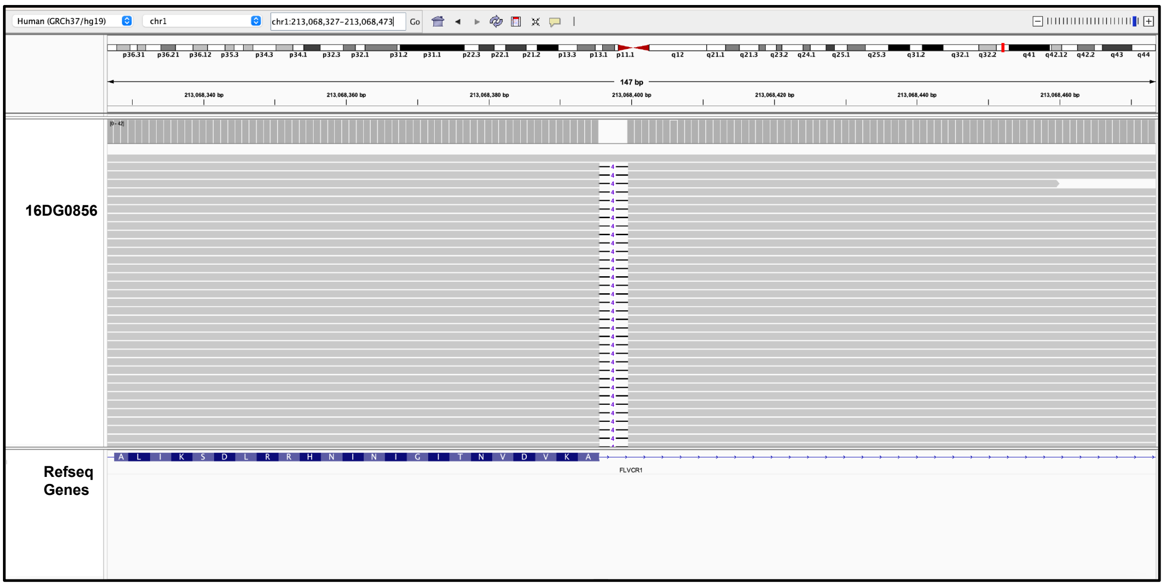

FLVCR1
NM_014053.4:c.1593+5_1593+8del

H

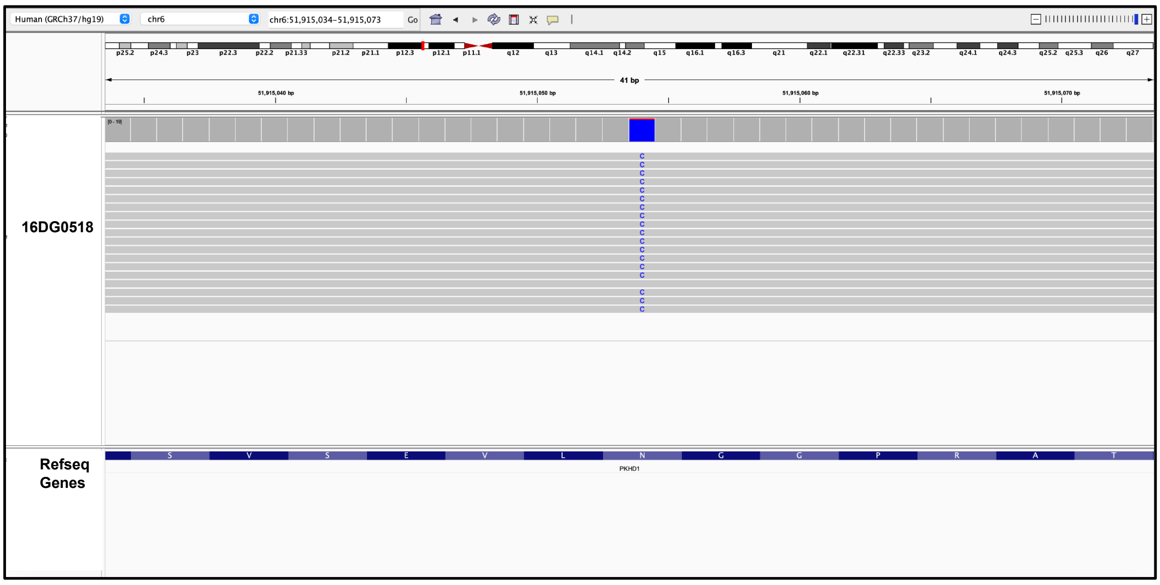

PKHD1
NM_138694.4:c.2180A>G;p.(Asn727Ser)

I

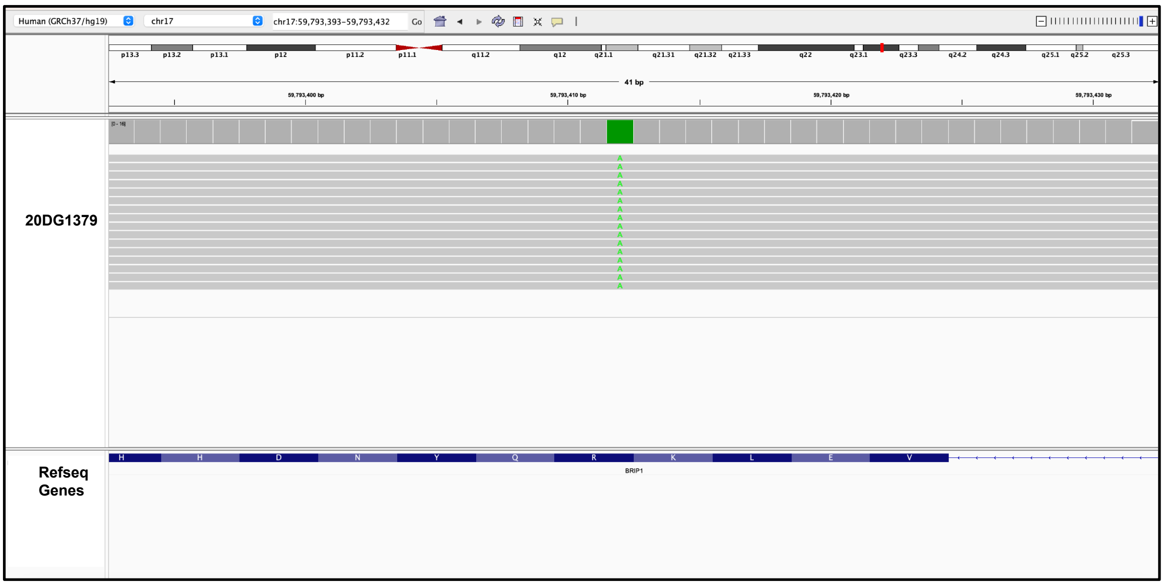

BRIP1
NM_032043.3:c.2392C>T;p.(Arg798*)

J

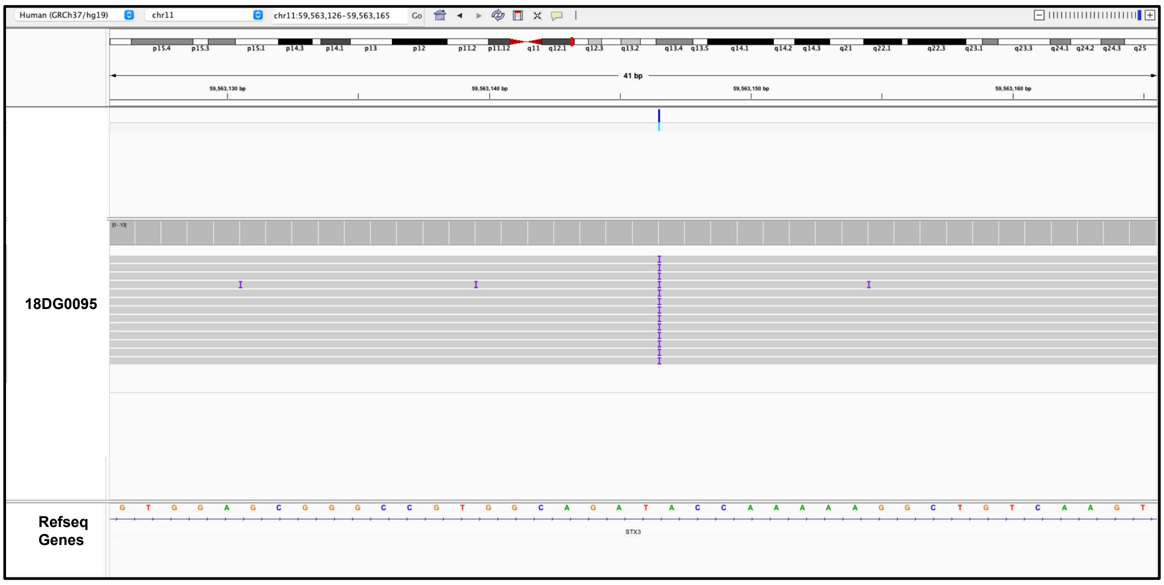

STX3
ENST00000437946.2:c.455dup;p.(Asp152Glufs*11)

K

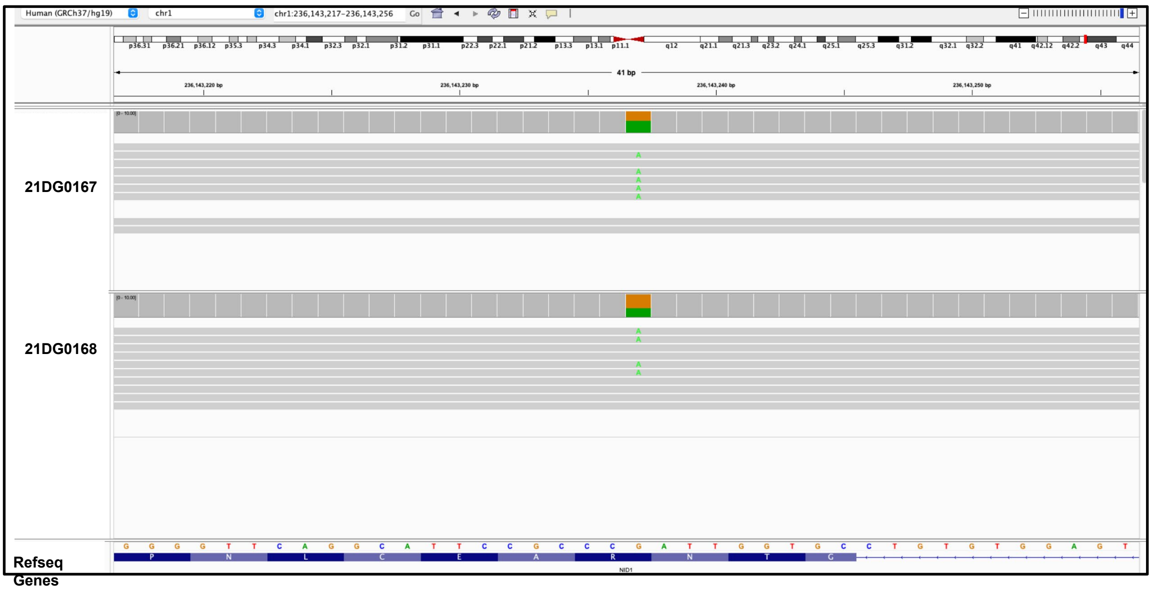

NID1
NM_002508.3:c.3394C>T;p.(Arg1132Trp)

L

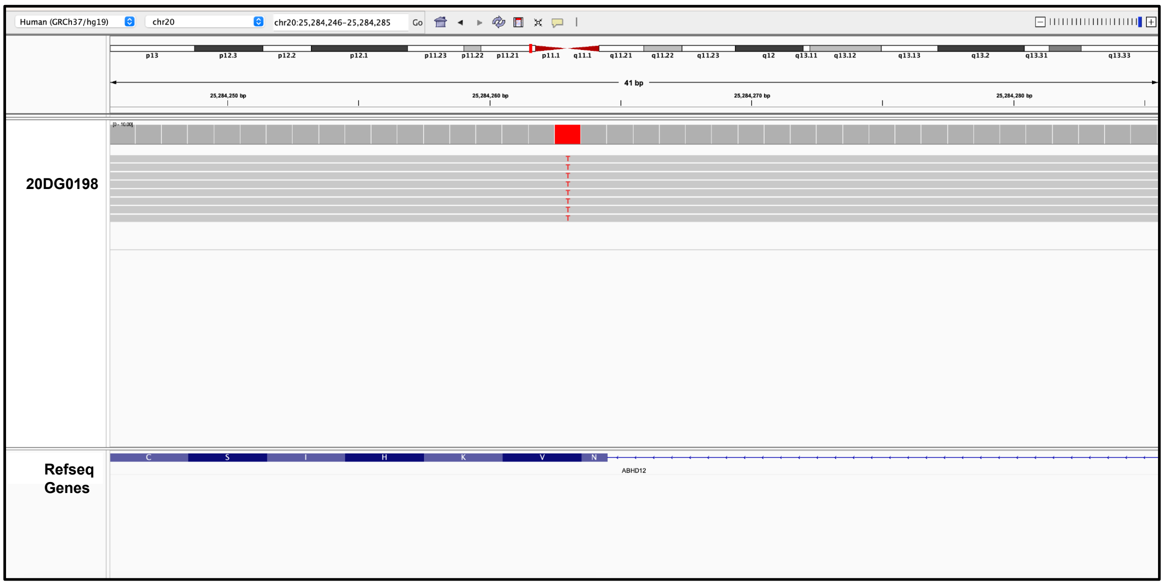

ABHD12
NM_001042472.3:c.952G>A;p.(Val318Met)

M

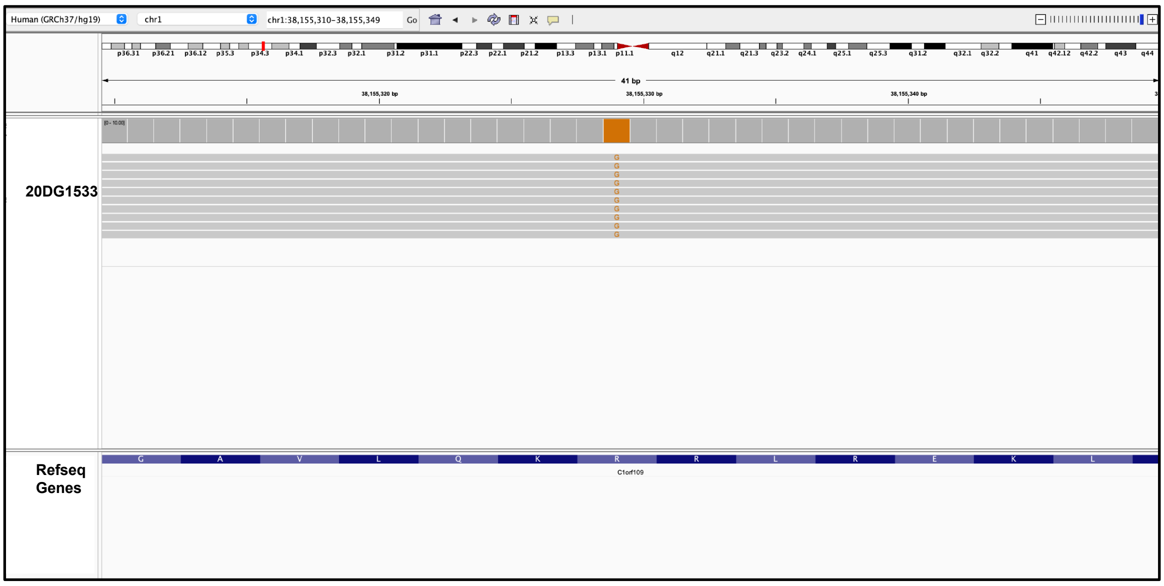

C1orf109
NM_001350767.2:c.224G>C;p.(Arg75Pro)

Fig. S1: Functional validation of *TYMS*, *SNAP91*, and *SLC4A4* variants.

A) Cell viability assay performed by treating fibroblast cells from patient (14DG1582) and two independent controls with 5-FU for 24hrs. Cell viability was measured using crystal violate and showed increase sensitivity of patient cells compared to control. Error bars denote standard deviation of 3 experiments. B and C) HEK293 cells were transfected with pCMV-RBGT1-SGFP-purified expression-ready linear constructs produced by PCR using (B) or (C) strategy as explained in Materials and Methods for 4 hours. Data are Mean +/- SEM of fluorescence intensity of the reporter activity. Data are Mean ± SEM. **$p < 0.01$ .D and E) Transmission electron microscope images of fibroblast cells from patient (14DG2098) and control (D) as well as LCLs from patients (14DG2102, 14DG2107, and 17DG0429) and control (E). Control images showed widespread vesicles and of numerous sizes compared to patient cells which had irregularly shaped inclusions that were smaller in numbers compared to control.

Fig. S2: Comparing variant detection using different depths on the Pacific Bioscience Sequel IIe platform.

A-D) Comparing the detection power when using different depths by employing the number of SNVs and indels detected as a proxy.

Fig. S3: PacBio data describing variants highlighted in this study.

A-M) IGV screenshots of variants in families discussed in this study.

Table S1: Sequences of primers used for the cloning-free reporter assay.

SLC4A4/-53-F	5´- CGC GGC CCC CCA GCC TCC AAC CCC GGT AGG CGT GTA CGG TG-3´
SLCA4A/-53M-F	5´- CGC GGC CCC CCA GCC TCC AAC CCT GGT AGG CGT GTA CGG TG-3´
SLC4A4/-53C-F	5´- CCA GCC TCC AAC CCC GGC GGC GCG CGG TAG GCG TGT ACG GTG-3´
SLC4A4/-53C/M-F	5´- CCA GCC TCC AAC CCT GGC GGC GCG CGG TAG GCG TGT ACG GTG-3´
Universal reverse primer	5´- CCA TAG AGC CCA CCG CAT G-3´

Table S2: Coverage statistics for PacBio-sequenced samples.

No	Sample ID	HiFi yield (Gb)	Coverage	Remarks
1	13DG1861	25.95	31	Trio Samples
	13DG1861	25.42		
	13DG1861	25.62		
	13DG1861	25.79		
2	16DG0856	29.30	53	
	16DG0856	25.89		
	16DG0856	25.60		
	16DG0856	30.66		
	16DG0856	32.31		
	16DG0856	29.67		
3	13DG1862	29.89	35	
	13DG1862	28.31		
	13DG1862	28.35		
	13DG1862	28.14		
4	17DG1097	35.61	11	
5	20DG0778	41.18	12	
6	19DG1210	28.40	9	
7	19DG1417	35.92	11	
8	15DG0371	32.17	10	
9	20DG1533	36.32	11	
10	20DG0198	37.10	11	
11	21DG0168	33.36	10	
12	21DG0319	27.18	8	
13	20DG0785	34.05	10	
14	17DG0832	33.96	10	
15	18DG0734	22.67	7	
16	22DG0245	33.75	10	
17	14DG1544	32.21	10	
18	18DG0095	33.53	10	
19	10DG0792	34.59	10	
20	14DG2098	38.59	12	
21	20DG0872	36.07	11	
22	14DG1582	37.53	11	
23	18DG0922	32.82	10	
24	21DG0167	39.41	12	
25	16DG0518	40.97	12	
26	21DG0734	43.51	13	
27	17DG0143	38.16	12	
28	08DG-00413	34.31	10	
29	20DG0235	36.79	11	
30	15DG0056	30.57	9	
31	14DG0876	21.10	6	
32	14DG1854	27.70	8	
33	15DG0960	21.13	6	
34	18DG0147	23.15	7	
35	20DG0820	43.80	13	
36	20DG0192	35.33	11	
37	20DG1379	34.01	10	
38	14DG1422	25.41	19	
	14DG1422	38.15		

Table S3: List of variants identified within ROHs which were ultimately excluded.

Family ID	Case ID	ROHs	Candidate Gene	Candidate Variant	Zygosity	Other candidates identified	Reason for proposing the causal variant
F4386	14DG1582	chr4:5505048-15467040, chr7:1181601-6535517, chr7:127850700-142458500, chr9:135745300-141077300, chr10:9773575-63186690, chr18:565414-6742958, and chr20:52932270-62906510	*TYMS*	NM_001071.4:c.455-2073ins of 270bp	Homozygous	Homozygous variant in *MRPL41* (NM_032477.3:c.362C>T;p.(Pro121Leu))	The missense variant in *MRPL41* failed segregation. Compelling biology, RT-qPCR results showed significant reduction in *TYMS* expression and cell viability assay data supporting the pathogenicity of the *TYMS* variant
F6404	20DG0785	chr2:224170800-233899400, chr2:237392500-243007900, chr3:104558700-133942900, chr4:7153211-15043740, chr7:37831300-45227590, chr7:79766350-106283800, chr9:98068780-103676700, chr9:107360800-109541500, chr11:14663580-22120650, chr11:115757900-120275200, and chr15:32018730-46564510	*STK25*	NM_001370694.2:c.2178+83del of 184bp (in *ANO7*)	Homozygous	No other candidates identified	Compelling biology and RT-qPCR data showed significant reduction in STK25 expression
F3981	17DG1097	No ROHs	*RP1L1*	NM_178857.6:c.4026_4027insACAGAAGAAGGGCTGCAAGAAGAGGGGGTGCAGTTAGAGGAAACTAAAACAGAAGAAGGGCTGCAAGAAGAGGGGGTGCAGTTAGAGGAAACTAAAACAGAAGAAGGGCTGCAAGAAGAGGGGGTGCAGTTAGAGGGGACTAAA;p.Glu1343delinsThrGluGluGlyLeuGlnGluGluGlyValGlnLeuGluGluThrLysThrGluGluGlyLeuGlnGluGluGlyValGlnLeuGluGluThrLysThrGluGluGlyLeuGlnGluGluGlyValGlnLeuGluGlyThrLysGlu and NM_178857.6:c.3970_3971insGGACTAAAGTAATAGAAGGGCTGCAAGAAGAGAGGGTGCAGTTAGAGG;p.Glu1324delinsGlyThrLysValIleGluGlyLeuGlnGluGluArgValGlnLeuGluGlu	Compound heterozygous	No other candidates identified	Known gene explaining the phenotype
F7974	20DG0235	chr4:59009-24590070, chr4:38306260-90884090, chr4:110881900-131431600, chr4:137887100-145343600, and chr17:3686003-5644253	*SLC4A4*	NM_001134742.2:c.-145C>T	Homozygous	No other candidates identified	Known gene explaining the phenotype and functional validation by RT-qPCR and reporter assay
F4591	14DG2098	Chr6: 80,016,660-86,734,460	*SNAP91*	NM_001242792.1:c.766-4799T>C	Homozygous	No other candidates identified	Compelling mouse model and functional validation by RT-qPCR and TEM imaging
F5927	17DG0832	chr6:21249760-30488200 and chr6:32645530-35107170	*LEMD2*	NM_181336.4:c.1011-469_1011-450del	Homozygous	chr6:33746614-33746633 (homozygous intronic deletion of *IP6K3*)	The intronic variant in *IP6K3* did not impact the gene's expression. Compelling biology and functional validation by RT-qPCR and nuclear morphology investigation supporting the pathogenicity of the *LEMD2* variant.
F3612	16DG0856	chr1:210068000-232432600, chr11:695,795-1,082,938, chr6:7034459-9587035, chr6:114448600-116354600, and chr11:6562742-8737724	*FLVCR1*	NM_014053.4:c.1593+5_1593+8del	Homozygous	chr11:881509-881656 (148bp homozygous intronic deletion in *CHID1*)	The intronic variant in *CHID1* did not impact the gene's expression. Compelling biology, mouse model, ongoing international cohort with compatible phenotypes and RT-PCR experiment supporting the pathogenicity of the *FLVCR1* variant.
F5543	16DG0518	Multiple ROHs	*PKHD1*	NM_138694.4:c.2180A>G;p.(Asn727Ser)	Homozygous	No other candidates identified	Known gene explaining the phenotype
F5349	20DG1379	chr12:17650460-20029510, chr15:42570720-45225350, chr16:66569180-68225510, chr17:34819190-36049820, and chr21:22060530-22810770	*BRIP1*	NM_032043.3:c.2392C>T;p.(Arg798*)	Homozygous	No other candidates identified	Known gene explaining the phenotype
F5993	18DG0095	chr6:36171210-37049770, chr6:39926300-44092130, chr6:46761880-49617860, chr8:22109110-23404770, chr9:79371210-81365970, and chr11:58331310-61616010	*STX3*	NM_004177.5:c.786+190dup (ENST00000437946.2:c.455dup;p.(Asp152Glufs*11))	Homozygous	No other candidates identified	Founder variant in a known gene explaining the phenotype
F8602	21DG0167 and 21DG0168	Multiple ROHs	*NID1*	NM_002508.3:c.3394C>T;p.(Arg1132Trp)	Heterozygous in parents	No other candidates identified	Compelling biology and a previously published family with presumed neonatal stroke (PMID: 25558065)
F7887	20DG0198	chr2:116086900-155366000, chr3:63143710-76401520, chr4:134251200-174860000, chr5:33457620-67457160, chr5:165136400-180698100, chr6:56010890-63634670, chr7:117308400-121368900, chr7:132716500-135928100, chr10:108349900-123762400, chr11:23699670-97753120, chr15:55665090-79411260, chr16:78641740-84779100, chr17:6487141-12923990, and chr20:15661000-35316880	*ABHD12*	NM_001042472.3:c.952G>A;p.(Val318Met)	Homozygous	chr4:170066984-170067056 (72bp homozygous deletion intronic of *SH3RF1*)	The intronic variant in *SH3RF1* did not impact the gene's expression. Compelling biology and in silico prediction supporting the pathogenicity of *ABHD12* variant
F8544	20DG1533	Multiple ROHs	*C1orf109*	NM_001350767.2:c.224G>C;p.(Arg75Pro)	Homozygous	No other candidates identified	Compelling mouse model and ongoing international cohort with compatible phenotypes
F6440	19DG1417	chr9:31537680-79306780	*CHMP5*	Duplication affecting CHMP5 (the coordinates for the gene chr9:33,264,877-33,282,067)	Homozygous	No other candidates identified	Compelling mouse model, cloning and RTPCR experiments confirming the truncating nature of the intragenic duplication
F8280	20DG0820	Multiple ROHs	SHFM3 locus	(10:102,950,203_103,472,860)x3	Heterozygous	No other candidates identified	Known locus explaining the phenotype

Table S4: Detailed listing of the study cohort.

Pedigree name	PacBio outcome	DG ID number	Disease Name	MIM #	Related-ness between parents	Gene	Variant	Zygosity	Phenotype	HGMD accession	ClinGen classification
F4386	Candidate variant identified	14DG1582	*TYMS*-related lactic acidosis	NA	First cousins	*TYMS*	NM_001071.4:c.455-2073ins of 270bp	Homozygous	Increased serum lactate; Encephalopathy; Abnormal breathing; Metabolic acidosis; Widened subarachnoid space; Focal T2 hyperintense brainstem lesion		Moderate_Variant evidence (2); Segregation evidence (3); I Experimental Evidence (2)
F6404	Candidate variant identified	20DG0785	*STK25*-related neurodevelopmental disorder	NA	First cousins	*STK25*	NM_001370694.2:c.2178+83del of 184bp (in *ANO7*)	Homozygous	Hearing impairment; Microcephaly; Short stature; Decreased body weight; Infra-orbital crease; Capillary hemangiomas (flat); Hypertonia; Failure to thrive		Moderate_Variant evidence (1); Segregation evidence (1); Animal model (2); Experimental Evidence (2)
F3981	Candidate variant identified	17DG1097	*Retinitis pigmentosa 88*	618826	Non-consanguineous	*RP1L1*	NM_178857.6:c.4026_4027ins[144];p.Glu1343delins[49] and NM_178857.6:c.3970_3971ins[48];p.Glu1324delins[17]	Compound heterozygous	Leber congenital amaurosis		NA (known gene)
F7974	Candidate variant identified	20DG0235	*SLC4A4*-related band keratopathy	NA	First cousins	*SLC4A4*	NM_001134742.2:c.-145C>T	Homozygous	Band keratopathy; Glaucoma; Corneal calcification; Choroidal detachment		NA (known gene)
F4591	Candidate variant identified	14DG2098	*SNAP91*-related microcephalic primordial dwarfism	NA	First cousins	*SNAP91*	NM_001242792.1:c.766-4799T>C	Homozygous	Dwarfism; Microcephaly; Global developmental delay; Spastic tetraparesis; Gastroesophageal reflux; Failure to thrive; Abnormal facial shape; Deeply set eye; Prominent nose; Hypoplasia of dental enamel; High palate; Abnormal sternum morphology; Brain atrophy (mild); Interhemispheric cyst (small)		Moderate_Variant evidence (1); Segregation evidence (4); Animal model (2); Experimental Evidence (1)
F5927	Candidate variant identified	17DG0832	*LEMD2*-related Neurodevelopmental disorder	NA	Same tribe	*LEMD2*	NM_181336.4:c.1011-469_1011-450del	Homozygous	Microcephaly; Plagiocephaly; Abnormal number of hair whorls; Anteverted nares; Microtia; Deep palmar crease; Slow saccadic eye movements; Heart murmur; Distal arthrogryposis; Adductor longus contractures; Increased muscle tone; Global developmental delay; Failure to thrive; Diffuse white matter abnormalities; Hip dysplasia; Dilatation of the renal pelvis; Mildly echogenic liver		Moderate_Variant evidence (1); Segregation evidence (4); Animal model (2); Experimental Evidence (1)
F3612	Candidate variant identified	16DG0856	*FLVCR1*-related Diamond-Blackfan anemia	NA	First cousins	*FLVCR1*	NM_014053.4:c.1593+5_1593+8del	Homozygous	Microcephaly; Intrauterine growth retardation; Micrognathia; Malar hypoplasia; Low-set ears; Wide nasal bridge; Cleft palate; Short neck; Dysplastic radii; Agenesis of the right thumb, Hypoplastic left thumb; Clinodactyly; Joint contractures	CD144017	Moderate_Variant evidence (2); Segregation evidence (2); Animal model (4); Experimental Evidence (2)
F5543	Candidate variant identified	16DG0518	*Polycystic kidney disease 4*	263200	Second cousins	*PKHD1*	NM_138694.4:c.2180A>G;p.(Asn727Ser)	Homozygous	Bilateral enlarged echogenic kidneys; Anhydramnios; Narrow chest; Flat nose; Rocker bottom feet; Distended abdomen; Intra-uterine fetal death	CM054802	NA (known gene)
F5349	Candidate variant identified	20DG1379	Fanconi anemia, complementation group J	609054	First cousins	*BRIP1*	NM_032043.3:c.2392C>T;p.(Arg798*)	Homozygous	Patent foramen ovale; PDA (large); Growth delayHypertelorism; Concave nasal ridge; Low-set ears; Posteriorly rotated ears; Ventriculomegaly; Intrauterine growth retardation; 2-3 toe syndactyly; Absent radius (bilateral); Hypoplasia of the ulna; Absent thumb (bilateral); Unilateral renal agenesis; Anal atresia	CM053140	NA (known gene)
F5993	Candidate variant identified	18DG0095	*STX3*-related retinal dystrophy	NA	First cousins	*STX3*	NM_004177.5:c.786+190dup (ENST00000437946.2:c.455dup; p.(Asp152Glufs*11))	Homozygous	Retinal dystrophy; Abnormality of retinal pigmentation		NA (known gene)
F8602	Candidate variant identified	21DG0167 and 21DG0168	*NID1*-related vein of Galen malformation	NA	Same tribe	*NID1*	NM_002508.3:c.3394C>T;p.(Arg1132Trp)	Heterozygous	Parents of a deceased neonate with: Vein of Galen aneurysmal malformation; PDA; Cardiomegaly; Retrograde flow in the descending aorta; Dilated superior vena cava; Right ventricular dilatation; Respiratory failure; Bone marrow hypocellularity; Hypotension; Hyponatremia; Metabolic acidosis;		Moderate_Variant evidence (2); Segregation evidence (3); Animal model (2)
F7887	Candidate variant identified	20DG0198	*ABHD12*-related developmental regression	NA	First cousins	*ABHD12*	NM_001042472.3:c.952G>A;p.(Val318Met)	Homozygous	Global developmental delay; Developmental regression; Ear pain; Generalized hypotonia; Hyporeflexia		NA (known gene)
F8544	Candidate variant identified	20DG1533	*C1orf109*-related neurodevelopmental disease	NA	Same tribe	*C1orf109*	NM_001350767.2:c.224G>C;p.(Arg75Pro)	Homozygous	Global brain atrophy; Global developmental delay; Spastic tetraplegia; Seizure; Blindness; Hearing impairment; Microcephaly; Upper and lower limb spasticity; Nystagmus; Flexion contracture		Moderate_Variant evidence (2); Segregation evidence (4.5); Experimental Evidence (2)
F6440	Candidate variant identified	19DG1417	*CHMP5*-relalated Neurodevelopmental disorder	NA	First cousins	*CHMP5*	Duplication affecting CHMP5 (the coordinates for the gene chr9:33,264,877-33,282,067)	Homozygous	Global developmental delay; Seizures; Microcephaly; Short stature; Decreased body weight; Muscular hypotonia of the trunk; Spasticity; Clonus; Scoliosis; Hirsutism		Moderate_Variant evidence (2); Segregation evidence (3); Experimental Evidence (2)
F8280	Candidate variant identified	20DG0820	SHFM3	246560	Double first cousins	SHFM3 locus	(10:102,950,203_103,472,860)x3	Heterozygous	Abnormality of limbs; Oligodactyly		NA (known locus)
F141	Unsolved	08DG-00413	*OTX2*-related infertility and retinitis pigmentosa	NA	First cousins	*OTX2*	NM_001270525.2:r.98_273del;p.(Pro34Metfs*3)	Homozygous	Rod-cone dystrophy; Infertility		Moderate_Variant evidence (2) ; Segregation evidence (5); Experimental Evidence (2)

Pedigree name	PacBio outcome	DG ID number	Disease Name	MIM #	Related-ness between parents	Gene	Variant	Zygosity	Phenotype	HGMD accession	ClinGen classification
F1070	Unsolved	10DG0792	*Congenital lipodystrophy*		First cousins				Congenital generalized lipodystrophy		
F4206	Unsolved	14DG0876	*Sensory and motor neuropathy*		Non-consanguineous				Sensorimotor neuropathy		
F4351	Unsolved	14DG1422	*Primordial dwarfism*		Consanguineous				Short stature; Intellectual disability; Severe failure to thrive		
F4379	Unsolved	14DG1544	*Progressive osteolysis*		Second cousins				Progressive osteolysis		
F4424	Unsolved	14DG1854	*Neurodevelopmental disorder*		First cousins				Intellectual disability; Long face; Mandibular prognathia; Broad-based gait; Dysdiadochokinesis		
F4697	Unsolved	15DG0056	*Short-rib thoracic dysplasia with polydactyly*		First cousins				Narrow chest; Dysmorphic facies; Prominent occiput; Short extremities; Polydactyly; Nail hypoplasia		
F4808	Unsolved	15DG0371	*Neurodevelopmental disorder*		First cousins				Abnormal facial shape; Triangular face; Depressed nasal bridge; Small and pointed chin; High arched palate; Small low set ears; Conical shaped incisors; Single palmar crease; Clinodactyly; Spina bifida occulta at LI; Bilateral sensorineural hearing loss; Global developmental delay		
F5016	Unsolved	15DG0960	*Adams-Oliver syndrome-like*		First cousins				Renovascular hypertension; Distal limb reduction anomalies; Brachydactyly; Specific learning disability; Clinodactyly of the 5th finger; Tapered finger; 2-3 toe syndactyly; Tapered toe; Short distal phalanx of finger; Sandal gap; Plantar crease between first and second toes; Short 5th toe; Short hallux; Small nail; 2-4 toe cutaneous syndactyly; Absent distal phalanges		
F5638	Unsolved	17DG0143	*Arthrogryposis multiplex*		First cousins				Arthrogryposis multiplex congenita		
F6001	Unsolved	18DG0147	*Intellectual disability and epilepsy*		First cousins				Intellectual disability; Seizures; Global developmental delay; Alopecia of scalp; Delayed speech and language development; Hyperactivity		
F6204	Unsolved	18DG0734	*Neurodevelopmental disorder*		Non-consanguineous				Seizures; Hemiparesis; Dilation of lateral ventricles; Hyperreflexia; Lower limb muscle weakness; Upper limb muscle weakness		
F6470	Unsolved	18DG0922	*Crohn's Disease*		First cousins				Crohn's disease		
F6859	Unsolved	19DG1210	*Primary ciliary dyskinesia*		First cousins				Dextrocardia; Abnormal heart morphology; Atrial septal defect; Gastroesophageal reflux; Asthma; Failure to thrive; Chronic constipation; Vomiting; Recurrent gastroenteritis; Polysplenia; Global developmental delay; Ciliary dyskinesia; Cough; Hypotonia; Recurrent lower respiratory tract infections		
F7886	Unsolved	20DG0192	*Neurodevelopmental disorder*		Second cousins				Attention deficit hyperactivity disorder; Autism; Intellectual disability; Delayed speech and language development; Obesity; Micropenis; Abnormal facial shape; Downslanted palpebral fissures; Thick lower lip vermilion; Widely spaced teeth		
F8283	Unsolved	20DG0778	*Striae distense*		Double first cousin				Striae distensae; Cutis laxa; Myopia; Localized skin lesion; Asymmetry of the breasts; Hypermelanotic macule; Premature skin wrinkling		
F8329	Unsolved	20DG0872	Polycystic kidney disease		First cousins				Polycystic kidney dysplasia; Hepatic fibrosis		
F8644	Unsolved	21DG0319	Joubert syndrome		Non-consanguineous				Global developmental delay; Hypotonia; Abnormal facial shape; Hypertelorism; Flat occiput; Cutaneous lesion Brisk reflexes; Molar tooth sign on MRI		
F4275	Unsolved	21DG0734	Jeune Syndrome		First cousins				Skeletal dysplasia; Mesomelic short stature; Lower thoracic interpediculate narrowness; Narrow greater sciatic notch; Polydactyly; Abnormality of the metacarpal bones; Abnormal metatarsal morphology; Abnormal finger phalanx morphology; Secundum atrial septal defect; Patent foramen ovale; Respiratory distress; Bell-shaped thorax; Echogenic fetal bowel; Fetal pyelectasis; Short long bone; Hand clenching; Abnormal foot morphology		

Table S5: List of transcription factors predicted to bind to the deleted region in F6404 with JASPER confidence scores.

TF list	p-value
PATZ1	10^{-7}
PLAG1	
KLF15	10^{-5}_ 10^{-6}
SP2	
KLF14	
ZNF320	
PRDM9	
ZNF610	
EGR1	
SP3	
KLF16	
ZNF148	
SP1	
KLF1	
SP4	
KLF12	
KLF10	
KLF7	
KLF5	
ZNF740	
RARA::RXRG	10^{-4}
ZNF530	
TFAP2A	
CTCFL	
ZNF93	
TFAP4::ETV1	
NR1I2	
ZIC5	
Zic1::Zic2	
EGR2	
ZBTB14	
Nrf1	
EGR3	
SP9	
ZNF343	
VEZF1	
KLF11	
KLF4	
KLF2	
KLF3	
KLF6	
MAZ	
INSM1	
ZNF454	
SP8	
ZNF281	
E2F6	
ZIC1	
GLIS2	
EGR4	
Wt1	
ZNF701	
NR2C2	
